# Mild Deoxygenation of Aromatic Ketones and Aldehydes over Pd/C Using Polymethylhydrosiloxane as the Reducing Agent**

**DOI:** 10.1002/anie.201411059

**Published:** 2015-02-26

**Authors:** Alexey Volkov, Karl P J Gustafson, Cheuk-Wai Tai, Oscar Verho, Jan-E Bäckvall, Hans Adolfsson

**Affiliations:** Department of Organic Chemistry, Stockholm UniversitySE-106 91, Stockholm (Sweden); Department of Materials and Environmental Chemistry, Arrhenius LaboratoryStockholm University, SE-106 91, Stockholm (Sweden)

**Keywords:** deoxygenation, heterogeneous catalysis, ketones, palladium, silanes

## Abstract

Herein, a practical and mild method for the deoxygenation of a wide range of benzylic aldehydes and ketones is described, which utilizes heterogeneous Pd/C as the catalyst together with the green hydride source, polymethylhydrosiloxane. The developed catalytic protocol is scalable and robust, as exemplified by the deoxygenation of ethyl vanillin, which was performed on a 30 mmol scale in an open-to-air setup using only 0.085 mol % Pd/C catalyst to furnish the corresponding deoxygenated product in 93 % yield within 3 hours at room temperature. Furthermore, the Pd/C catalyst was shown to be recyclable up to 6 times without any observable decrease in efficiency and it exhibited low metal leaching under the reaction conditions.

Reductive deoxygenation of aldehydes and ketones to the corresponding saturated compounds has attracted considerable attention given its many applications in fine-chemical synthesis[1] and biofuel production.[2] Unfortunately, classical methods for the deoxygenation of carbonyl compounds, such as those based on either the Barton–McCombie,[3] Clemmensen,[4] or Wolff–Kishner[5] methodologies, are generally associated with harsh reaction conditions, the use of stoichiometric amounts of toxic reagents, and poor functional-group tolerance. Later developments led to mild stoichiometric protocols for the deoxygenation of carbonyl compounds, where different metal hydrides are used as reagents.[6] However, these methods generally suffer from unsatisfactory chemoselectivity, poor atom economy, and substantial byproduct formation, which complicate the workup and purification procedures.

Currently, there exist a number of catalytic protocols for the deoxygenation of carbonyls employing molecular hydrogen (H_2_).[7] Although, H_2_ constitutes the most atom-efficient and green reducing agent, its use is generally associated with high pressure, special equipment, and safety precautions to minimize the explosion risk.[8] Among the many hydride donors used in catalytic procedures,[9] silanes can be considered as one of the most attractive donors since they are generally cheap and easy to handle. Moreover, silanes usually allow the reduction to occur under milder reaction conditions and with higher chemoselectivity in comparison to other methods.[10] Of the many available silanes, polymethylhydrosiloxane (PMHS) is an attractive option as it demonstrates high air and moisture stability. PMHS is cost-effective and readily available, since it is generated in large quantities as a byproduct in the silicon industry.[11] To date, several transition-metal-based catalytic protocols employing PMHS as the reducing agent have been developed for the reduction of amides to amines,[12] as well as for the conversion of ketones, aldehydes, and esters either into alcohols[13] or to saturated alkanes.[14]

Although homogeneous systems for the deoxygenation of aromatic alcohols, aldehydes, and ketones with PMHS have been developed,[15,16] there exists no heterogeneous catalytic system for the deoxygenation of aromatic ketones and aldehydes under hydrosilylation conditions. Heterogeneous protocols are generally associated with several practical advantages, such as improved catalyst stability, simpler separation and purification procedures, possibility of catalyst recycling, and lower levels of metal impurities in the final product. Herein, we report on the first heterogeneous system for the deoxygenation of a wide range of aromatic carbonyl compounds with good chemoselectivity by utilizing low catalyst loading of commercially available palladium on carbon (Pd/C) and PMHS under mild reaction conditions.

For preliminary screening, the deoxygenation of 4-methoxyacetophenone (**1**) into 4-ethylanisole (**3**) was investigated in different solvents (see Table S1 in the Supporting Information). We identified that commercially available Pd/C (5 wt % according to commercial provider)[17] could function as an efficient catalyst for this deoxygenation reaction when PMHS is used as the hydride source. The solvent screening was carried out at 65 °C for over 16 hours, and this revealed that the reaction exhibited the highest efficiencies in alcohol solvents, while nonprotic solvents resulted in no or low conversions. The best results were observed in alcohols bearing long alkyl chains, and were ascribed to a better solubility of the polymeric PMHS in these solvents (Scheme 1). However, the reaction still exhibited satisfactory activity in MeOH, thus resulting in full conversion of the starting ketone **1** to alcohol **2** and deoxygenated product **3** in a ratio of 2:3. Therefore, to allow simple isolation procedures of the deoxygenated products with relatively low boiling points, the more volatile MeOH was used as the solvent for further experiments.

**Scheme 1:**
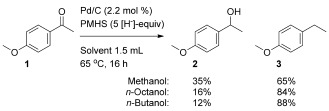
The deoxygenation of 4-methoxyacetophenone using Pd/C and PMHS in alcoholic solvents.

After the screening of solvents, a number of different silanes were evaluated as hydride donors for this reaction (Table 1), and none of the chosen silanes were found to outperform PMHS. In the case of trimethoxysilane, tetramethyldisiloxane (TMDS) and triethylsilane, lower yields of the deoxygenated product **3** were obtained (entries 2–4). Interestingly, the most reactive silane, phenylsilane, was found to give the lowest conversion into **3** (entry 5), thus emphasizing that the stability of the silane is more important than its activity for the overall efficiency of the reaction.

**Table 1:** Screening of silanes for the deoxygenation of compound 1. 
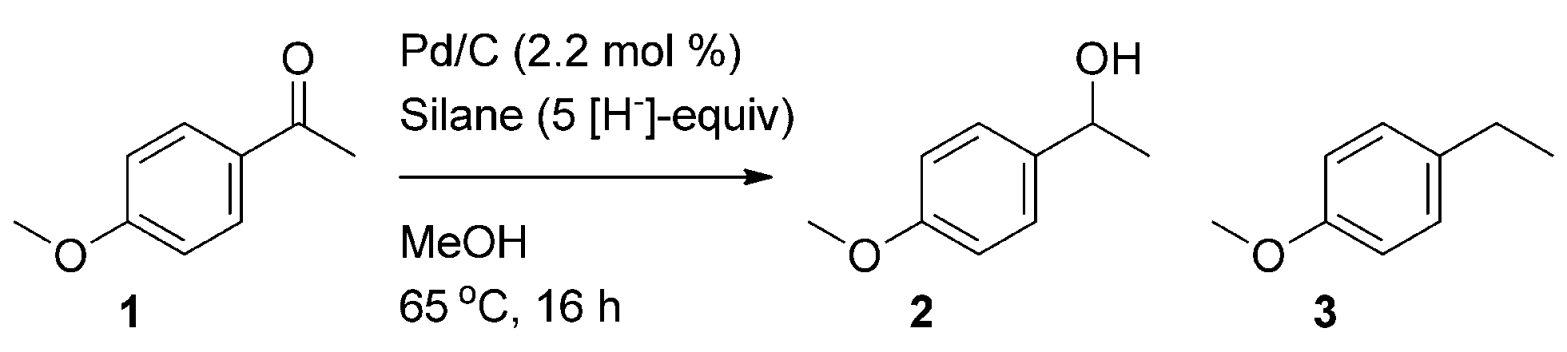

Entry[a]		Silane		Conversion [%][b]		
				1	2	3
1		PMHS		0	35	65
2		Et_3_SiH		0	67	33
3		(MeO)_3_SiH		0	50	50
4		TMDS		0	60	40
5		PhSiH_3_		0	87	13

[a] Unless otherwise noted, all reactions were carried out on a 0.5 mmol scale with 2.2 mol % Pd/C in 1.5 mL of a solvent with 5 equiv of PMHS.

[b] [b] Conversion of the starting compound was determined by ^1^H NMR spectroscopy. TMDS=1,1,3,3-tetramethyldisiloxane.

Having established MeOH and PMHS as the optimal solvent and silane, respectively, for this transformation, we next focused on improving the yield of the fully saturated product **3**. By monitoring the reactions in protic solvents with ^1^H NMR spectroscopy, it was established that the dehydration of the alcohol intermediate **2** constituted the rate-limiting step of the overall deoxygenation process. It was anticipated that protonation of the alcohol moiety would promote this dehydration step and lead to a more efficient deoxygenation reaction. Therefore, a number of acidic additives were screened and to our delight, we observed that addition of 5 mol % of hydrochloric acid (present in either aqueous solutions or in diethyl ether) or acetic acid led to excellent conversions of the model substrate **1** into **3** (Table 2, entries 1–3). Rahaim, Jr. et al. have previously demonstrated that chlorobenzene could function as a convenient acid equivalent which delivers hydrochloric acid in situ upon oxidative addition to palladium and subsequent reduction.[15] Gratifyingly, we observed full conversion of **1** into **3** when chlorobenzene was employed as an additive. The use of an acid additive also made it possible to decrease the reaction time to 2 hours and to run the reaction at room temperature (entries 4 and 5). Furthermore, it allowed a decrease of the catalyst loading to 0.4 mol % and the hydride source to three equivalents without affecting the efficiency of the reaction (entry 6). It was found that the support (C, SiO_2_, or Al_2_O_3_) for the heterogeneous Pd had no significant influence on the catalyst activity. In contrast, exchanging Pd for either Pt, Ru, or Rh on carbon was detrimental for the reaction outcome (see Table S2 in the Supporting Information).

**Table 2:** Influence of the catalytic amounts of additives on deoxygenation of the model substrate. 
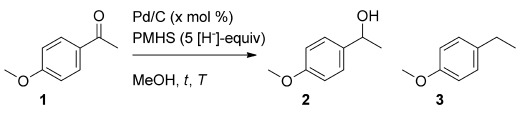

Entry[a]	Pd/C (mol %)	*T* [°C]	*t* [h]	Additive	Conversion [%][b]		
				(5 mol %)	1	2	3
1	2.2	65	16	AcOH	0	7	93
2	2.2	65	16	HCl 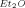 [c]	0	0	>95
3	2.2	65	16	HCl_w_[d]	0	0	>95
4	2.2	65	16	PhCl	0	0	>95
5	2.2	RT	2	PhCl	0	0	>95
6[e]	0.4	RT	2	PhCl	0	0	>95

[a] Unless otherwise noted, all reactions were carried out on a 0.5 mmol scale with 2.2 mol % Pd/C in 1.5 mL of a solvent with 5 equiv of PMHS.

[b] Conversion of the starting compound was determined by ^1^H NMR spectroscopy.

[c] 1 m HCl solution in Et_2_O.

[d] 37 % water solution of HCl.

[e] 3 [H^-^] equiv of the PMHS were used.

With an optimized protocol in hand for the deoxygenation of **1**, we investigated the scope of this method on a broad set of aromatic aldehydes and ketones (Table 3). As expected, electron-rich ketones and aldehydes were found to react faster than the electron-poor counterparts, and the corresponding deoxygenated products were obtained in good yields under the optimized reaction conditions (entries 4, 12, 15, and 16). For electron-neutral arenes, no apparent decrease in the rate of product formation could be observed (entries 1, 3, and 14). In certain cases, the steric hindrance around the carbonyl functionality called for either prolonged reaction times or a temperature of 40 °C to give satisfactory yields of the deoxygenated products (entries 13, 18, and 19). An important advantage of the present system over previously reported protocols is the fact that it also works for deactivated ketones and aldehydes bearing strong electron-withdrawing groups. As demonstrated for electron-deficient substrates, it was possible to obtain the corresponding deoxygenated products in high to excellent yields with only 0.4 mol % of Pd/C, although prolonged reaction times and a temperature of 40 °C were required (entries 2, 5–9, and 17). Moreover, the developed catalytic system proved to be compatible with several important functional groups such as esters, amides, amines, phenols, and carboxylic acids (entries 7, 9, 10, 16, 17, and 19). Unfortunately for 4-nitroacetophenone, the reduction of the nitro group was found to occur prior to the deoxygenation of the carbonyl group, thus resulting in the formation of 4-ethylaniline in 91 % yield (entry 11). Interestingly, this protocol could also be applied for the deoxygenation of acetylferrocene, thus giving ethylferrocene in 92 % yield upon isolation. The product is a compound which is used as a fuel additive in rocket propellants to promote the rate of ignition (entry 20).[18] Aliphatic ketones proved to be more difficult to reduce using the optimized protocol, as exemplified by the reaction of 2-decanone which resulted in 14 % conversion into the corresponding alcohol after 6 hours. However, this feature could be exploited for achieving selective deoxygenation of an aromatic carbonyl in the presence of an aliphatic one (entry 21).

**Table 3:** Palladium on carbon catalyzed deoxygenation of ketones and aldehydes under hydrosilylation conditions.

Entry[a]	Substrate	Product	*t* [h]	*T* [°C]	Yield [%]
	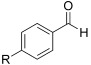	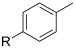			
1	R=H	R=H	2	RT	>95[b]
2	R=CF_3_	R=CF_3_	2	40	>95[b]
					
	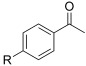	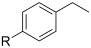			
3	R=H	R=H	2	RT	>95[b]
4	R=OMe	R=OMe	2	RT	>95[b]
5	R=F	R=F	6	40	>95[b]
6	R=CF_3_	R=CF_3_	18	40	>95[b]
7	R=C(O)OMe	R=C(O)OMe	14	40	>95[b]
8	R=C_6_H_5_	R=C_6_H_5_	4	40	99[c]
9	R=NHC(O)Me	R=NHC(O)Me	3	40	90[c]
10[d]	R=NH_2_	R=NH_2_	8	RT	>95[b]
11	R=NO_2_	R=NH_2_	16	40	91[be]
					
12	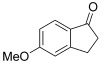	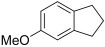	2	RT	96[c]
13	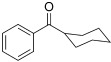	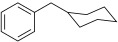	9	RT	88[c]
14	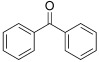	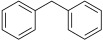	2	RT	99[c]
15	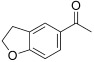	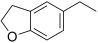	2	RT	87[c]
16	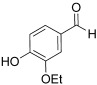	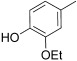	2	RT	92^[c,f]^
17	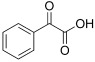	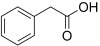	5	40	99[c]
18	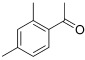	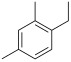	4	RT	>95[b]
19	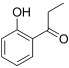	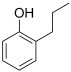	4	40	93[c]
20	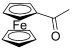	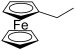	5	40	92[c]
21	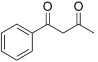	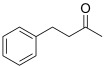	6	RT	80[c]

[a] Unless otherwise noted, all reactions were carried out on a 0.5 mmol scale with 0.4 mol % Pd/C and 5 mol % PhCl in 1.5 mL of MeOH with 3 equiv of PMHS.

[b] Yield was determined by NMR spectroscopy using 1,3,5-trimethoxybenzene as the internal standard.

[c] Yield of isolated product.

[d] The reaction was run with 5 equiv of PMHS.

[e] [e] Additional 3 equiv of PMHS were added to the reaction after 8 h. [f] The reaction was also performed on a 30 mmol scale and resulted in 93 % yield of the isolated product.

To evaluate the scalability of the present protocol, the deoxygenation of ethyl vanillin was performed on a 30 mmol scale in an open flask setup using 0.085 mol % Pd/C and 5 mol % HCl (aq) as acid additive (Table 3, entry 16). Gratifyingly, quantitative deoxygenation of the starting compound was observed already after 3 hours and the product could be isolated in 93 % yield (4.3 g).

When developing a heterogeneous protocol for practical use, it is of fundamental importance to establish the recyclability of the catalyst under the employed reaction conditions, as well as to determine the metal leaching. To investigate the stability of the Pd/C catalyst, it was recovered from the deoxygenation reaction of **1** by the use of centrifugation. It was washed with methanol three times, and subjected to a subsequent reaction cycle. To our delight, the catalyst showed excellent recyclability and could be reused six times without any noticeable loss of efficiency, even though TEM analysis of recycled catalyst revealed that the Pd particles had increased in size over multiple uses (see Figure S1–S6 in the Supporting Information). Interestingly, inspection of the particle size distribution of unused Pd/C revealed that it consisted of a large number of small and well-defined Pd nanoparticles in the size range of 0.8–2.4 nm. In contrast, the catalyst recovered after one and six reactions contained more distorted Pd nanoparticles predominately in the size range of 0.8–4.0 nm. XPS analysis showed that the surface of the unused catalyst is mainly composed of Pd^II^ (ca. 2/3 of total Pd signal), whereas the catalyst recovered after one cycle was predominant in the Pd^0^ state (ca. 90 % of the total Pd signal; see Figures S7 and S8). Gratifyingly, the Pd leaching was found to be very low in the reaction as determined by ICP-OES-analysis (0.6 ppm, 0.32 % of the total amount of Pd used).

As was mentioned above, H_2_ constitutes the most atom-efficient hydride source for the reduction reactions. Therefore, we were interested in comparing the efficiency of the current system to the corresponding hydrogen-based deoxygenation, in the presence of 5 mol % of aqueous HCl (Figure 1). The rates of the consumption of the ketone and the formations of alcohol intermediate and deoxygenated product were monitored by ^1^H NMR spectroscopy. The starting material was almost immediately converted into the corresponding alcohol under hydrosilylation conditions (Figure 1 a), in contrast to the reduction using H_2_, which progressed significantly slower (Figure 1 b). Moreover, the hydrosilylation protocol resulted in 90 % yield of deoxygenation product within 1 hour, whereas the hydrogenation reaction afforded only 11 % of deoxygenation product after the same amount of time.

**Figure 1:**
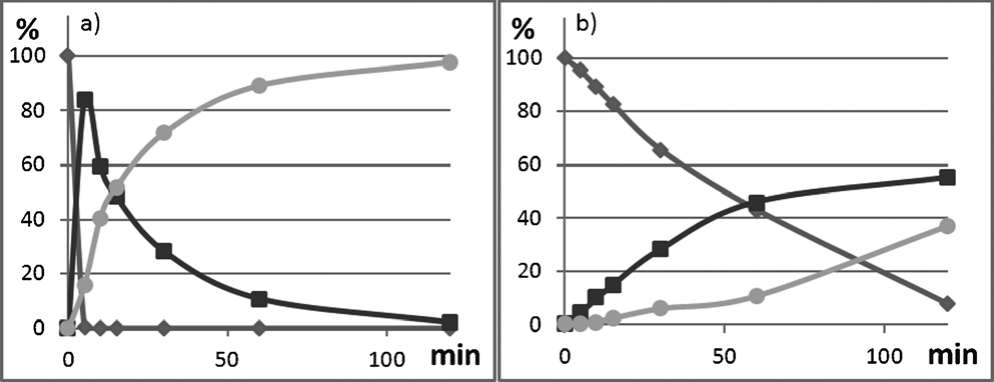
Comparison of the deoxygenation of the model substrate acetophenone (Table 1, entry 3) under hydrosilylation (a) and hydrogenation conditions (b). ketone, ▪ alcohol, • product.

The reaction proceeds in two steps, where the ketone is first reduced to the alcohol, followed by deoxygenation to form the saturated product (Scheme 2). Attempts to elucidate the identity of the reducing Pd species (i.e. PdH_2_ or PdH[Si]) in this catalytic reaction by ^1^H NMR spectroscopy proved unfruitful as the surface-generated Pd-H species and the deoxygenated product were both found to undergo a facile H/D exchange with CH_3_OD as the solvent under the employed reaction conditions.[19] In contrast, we could establish that the extent of deuterium incorporation remained constant when the solvent was changed from CH_3_OD to CD_3_OD, and indicates that the solvent does not act as a hydrogen donor in this reaction.

**Scheme 2:**
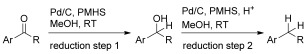
Deoxygenation of ketones and aldehydes as a two-step process.

In conclusion we have developed an efficient heterogeneous catalytic system for the deoxygenation of aromatic ketones and aldehydes bearing both electron-donating and electron-withdrawing groups. Moreover, this protocol was found to tolerate a wide range of functional groups, such as amides, amines, esters, carboxylic acids, aliphatic ketones, and phenols. Another advantage of this protocol is that it utilizes a cheap and environmentally benign reducing agent (PMHS) together with low loading of a readily available catalyst, palladium on carbon (Pd/C). With this procedure, electron-deficient aromatic ketones and aldehydes were, for the first time, efficiently deoxygenated to the corresponding alkyl arenes. Under the optimized reaction conditions, the Pd/C catalyst is recyclable up to six times without any loss in activity and it showed low leaching of Pd into solution. In addition, the procedure was scaled up to 30 mmol in an open-to-air setup, thus allowing the catalyst loading to be reduced to 0.085 mol % while maintaining an excellent yield of 2-ethoxy-4-methylphenol. Together, all these advantages make this catalytic protocol a green and highly economical process for the deoxygenation of ketones and aldehydes, and has the potential to be utilized in industrial settings.
