# Supplementary material for: Mild Deoxygenation of Aromatic Ketones and Aldehydes over Pd/C Using Polymethylhydrosiloxane as the Reducing Agent
Source: Angew Chem Int Ed Engl. 2015 Feb 26;54(17):5122–6. doi: 10.1002/anie.201411059 (PMC4471587; doi:10.1002/anie.201411059)
Supplement: Supplementary file 1 [file anie0054-5122-sd1.pdf]

Angewandte Chemie
Eine Zeitschrift der Gesellschaft Deutscher Chemiker

Supporting Information

Mild Deoxygenation of Aromatic Ketones and Aldehydes over Pd/C Using Polymethylhydrosiloxane as the Reducing Agent**

Alexey Volkov, Karl P. J. Gustafson, Cheuk-Wai Tai, Oscar Verho, Jan-E. Bäckvall,* and Hans Adolfsson**

anie_201411059_sm_miscellaneous_information.pdf

Table of content

General information 2
Solvent and catalyst screening 4
General procedure for catalytic deoxygenation of ketones and aldehydes. 5
Deuterium incorporation study 6
Characterization of unused and recycled Pd/C catalyst by STEM 8
Characterization of unused and recycled Pd/C catalyst by XPS.................................13
Recycling study of the Pd/C in the deoxygenation reaction 15
Not isolated products: 16
Isolated products: 17
Reference 20
Spectroscopic data. 21

General information

Unless otherwise noted, all materials were obtained from commercial suppliers and used without further purification. Flash chromatography was performed on an automated flash chromatography instrument using silica-based cartridges with UV detection for fraction collection. ^{1}H-NMR and ^{13}C-NMR were recorded on a Bruker Avance 400 MHz instrument. Chemical shifts in ^{1}H-NMR and ^{13}C-NMR are reported in ppm, relative to solvent peaks (^{1}H δ_H: $CDCl_3$ 7.26 or d^6-DMSO 2.50 and ^{13}C δ_C: $CDCl_3$ 77.0 or d^6-DMSO 39.5. The following abbreviations were used to explain multiplicities: s = singlet, bs = broad singlet, d = doublet, t = triplet, q = quartet, m = multiplet. MS data were obtained from GC-MS using EI detection.

The Pd/C catalyst used in these studies was purchased from Sigma-Aldrich (containing 5 wt% Pd according to the supplier). However, analysis of this catalyst batch by Inductively Coupled Plasma-Optical Emission Spectroscopy (ICP-OES, Medac Ltd, Analytical and Chemical Consultancy Services, United Kingdom) determined the actual Pd loading to 4.23 wt%. The catalyst loadings used in all reactions were based on a Pd loading of 5 wt%, and then corrected to reflect the true loading of 4.23 wt% Pd afterwards. The palladium leaching from the catalyst was determined by analyzing a liquid aliquot from a representative reaction by ICP-OES. The size and distribution of the Pd particles in the catalyst were determined by Scanning Transmission Electron Microscopy (STEM). The high-angle annular dark-field STEM (HAADF-STEM) images, also known as Z-contrast images, were taken at room temperature using a JEOL JEM-2100F field-emission microscope equipped with a JEOL ADF detector. The microscope was operated at 200 kV, and the probe size and camera length used were 0.20 nm and 8 cm, respectively. Characterization of the oxidation states of the Pd particles was done by XPS (X-Ray Photoelectron Spectroscopy)/ESCA (Electron Spectroscopy for Chemical Analysis) (SP Technical Research Institute of Sweden, SP Chemistry, Materials and Surfaces). XPS spectral data was recorded using a Kratos AXIS Ultra^{DLD} X-ray photoelectron spectrometer (Kratos Analytical, Manchester, UK). The samples were analyzed using a monochromatic Al X-ray source, analysis area was ca 1 mm^2 (most of the signal is from an area of 700 x 300 μm). The peaks were adjusted after the C 1s peak that was set to 284.4 eV as the reference.

The catalytic deoxygenation experiments were conducted on a 0.5 mmol scale in Biotage microwave tubes (2-5 mL) with a Teflon-coated magnetic stirring bar. All reactions were capped with a cap containing a septum and were run under ambient atmosphere.

Solvent and catalyst screening

Table S1. Solvent screening for the deoxygenation of compound **1**.

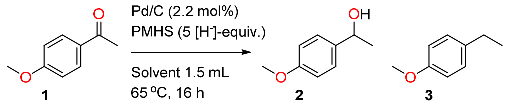

Entry[a]	Solvent	**1**[b]	**2**[b]	**3**[b]
1	THF	>95	0	0
2	Toluene	>95	0	0
3	EtOAc	>95	0	0
4	DCM	79	21	0
5	PEG	93	7	trace
6	n-Octanol	0	16	84
7	n-Butanol	0	12	88
8	Methanol	0	35	65

[a] Unless otherwise noted, all reactions were carried out on a 0.5 mmol scale with 2.2 mol % Pd/C in 1.5 mL of a solvent with 5 equiv. of PMHS; [b] Conversion of the starting compound was determined by ^{1}H NMR (%).

Table S2. Catalyst screening for the deoxygenation of compound **1**.

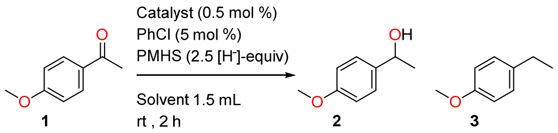

Entry[a]	Catalyst	**1**[b]	**2**[b]	**3**[b]
1	Pd on alumina 10 wt % (Aldrich)	0	10	90
2	Pd on MCF (SiO_2)	0	10	90
3	Pd on carbon 10 wt % (Acros)	0	0	>95
4	Pd on carbon 5 wt % (Lancaster)	0	3	>95
5	Pd on carbon 5 wt % (Aldrich)	0	0	>95
6	Pd on $CaCO_3$ 5 wt %, poisoned with lead (Aldrich)	>95	0	0
7[c]	Pt on carbon 5 wt % (Aldrich)	>95	0	0
8[c]	Ru on carbon 5wt % (Fluka)	95	5	0
9[c]	Rh on Carbon 5 wt % (Fluka)	>95	0	0

[a] Unless otherwise noted, all reactions were carried out on a 0.5 mmol scale with 0.5 mol % of catalyst in 1.5 mL of a solvent with 2.5 equiv of PMHS at rt for 2 h; [b] Conversion of the starting compound was determined by ^{1}H NMR (%); [c] 3 equiv of PMHS was used.

General procedure for catalytic deoxygenation of ketones and aldehydes.

The substrate aldehyde/ketone (0.5 mmol) and Pd/C (4.23 wt%, 5.3 mg, 0.0021 mmol Pd) were suspended in MeOH (1.5 mL) in a sealed microwave tube. To this was added chlorobenzene (5 μL, 0.025 mmol) and PMHS (0.09 mL, 3 – 5 equiv of [H^-]) under stirring. The reaction mixture was then allowed to stir at rt or 40 °C for the time given in Table 4. After completion, the reaction mixture was transferred to a round-bottomed flask and to this silica gel (2 g) and DCM (5 mL) were added. After mixing, the solvents were removed by evaporation under reduced pressure and the crude silica material was charged on a silica-based cartridge. The desired deoxygenated compounds were purified by flash chromatography using pentane/EtOAc (from 1:0 to 10:1) as the eluent. The pure deoxygenated products were analyzed by ^{1}H and ^{13}C-NMR, and the spectra are presented in the section "compound characterization".

For deoxygenated products with boiling point lower than 200 °C, the yield of the reaction was determined against 1,3,5-trimethoxybenzene (8.4 mg, 0.05 mmol), which was used as an internal standard. For yield determination against the internal standard, crude samples were withdrawn from the reaction solution at the end of the reaction, filtered through a celite plug, diluted with $CDCl_3$ and analyzed by ^{1}H-NMR.

Deuterium incorporation study

Deuterium incorporation was measured using ^{1}H NMR technique, comparing the integral of the protons at the benzylic position to aromatic and terminal methyl group protons. Isotopomeric ratio was determined using GC-MS. Percentage of deuterium incorporation is calculated from the theoretical maximum amount of deuterium atoms that can be introduced during the course of the reaction, *i.e.* in the ketone reduction to the alcohol, the latter can only contain one deuterium and this will constitute the theoretical maximum of 100% deuterium incorporation.

In an attempt to gain insights into the mechanism of this reaction, we conducted a series of deuterium labeling experiments using both 4-acetylbiphenyl and 1-(4-biphenylyl)-1-ethanol as the starting materials in CH_3OD, but unfortunately these studies where all complicated by various exchange processes.

Table S3.

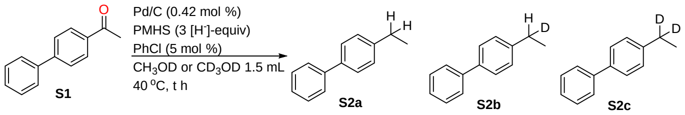

Entry[a]	Time	S2a %	S2b %	S2c %	D – incorporation
1	30 min	45	40	15	25 %
2	4 h	26	47	27	50 %

a) Unless otherwise noted, all reactions were carried out on a 0.5 mmol scale with 0.42 mol % Pd/C in 1.5 mL of MeOH with 3 equiv of PMHS.

Table S4.

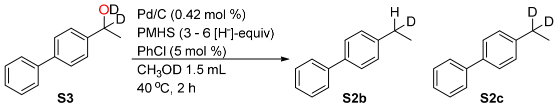

Entry[a]	Time	S2b %	S2c %	D - incorporation
1	2 h	50	50	50 %

a) Unless otherwise noted, all reactions were carried out on a 0.5 mmol scale with 0.42 mol % Pd/C in 1.5 mL of MeOH with 4 equiv of PMHS.

Table S5.

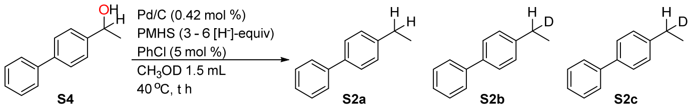

Entry[a]	Time	S2a %	S2b %	S2c %	D – incorporation
1	15 min	69	29	2	30 %
2	2 h	47	46	7	50 %
3	4 h	36	50	14	70 %
4[b]	15 min	-	-	-	70 %
5[b]	2 h	-	-	-	80 %

a) Unless otherwise noted, all reactions were carried out on a 0.5 mmol scale with 0.42 mol % Pd/C in 1.5 mL of MeOH with 3 equiv of PMHS; b) 0.42 mol % Pd/C, 5 mol% PhCl and 4 equiv of PMHS were stirred in 0.5 mL of MeOD for 15 min at 40 ˚C, starting alcohol **S4** was added to the reaction mixture as a solution in 1 mL of MeOD.

For instance, we observed that the deoxygenated product, 4-ethylbiphenyl, underwent significant post-catalytic exchange, which led to increased deuterium incorporation over time, making it difficult to fully distinguish the deuterium incorporated from the mechanistically-relevant pathway (Table S5). Therefore, it was decided to investigate the incorporation of deuterium at an early phase of the deoxygenation of 4-acetylbiphenyl (after 15 min). In this experiment, we could observe a deuterium incorporation of 25% in the two reduced products. However, when the catalyst was pretreated with PMHS in CH_3OD for 15 min prior to substrate addition, we could detect a significantly higher deuterium content of 70% after 15 min, together with the build-up of pressure that was indicative of in situ formation of hydrogen gas. This latter experiment provided strong support for a second exchange process, where the Pd-hydride species generated through the reaction undergo H/D-exchange with the solvent.

It can be envisioned that the intermediate Pd–H species located on the catalyst surface could undergo rapid exchange with the deuterated solvent that would lead to increased deuterium incorporation in the final product.

Characterization of unused and recycled Pd/C catalyst by STEM

To establish if the Pd/C underwent particle agglomeration upon consecutive usage, the unused and recycled catalysts (after first reaction and last cycle) were studied by HAADF-STEM and the particle size distributions were determined.

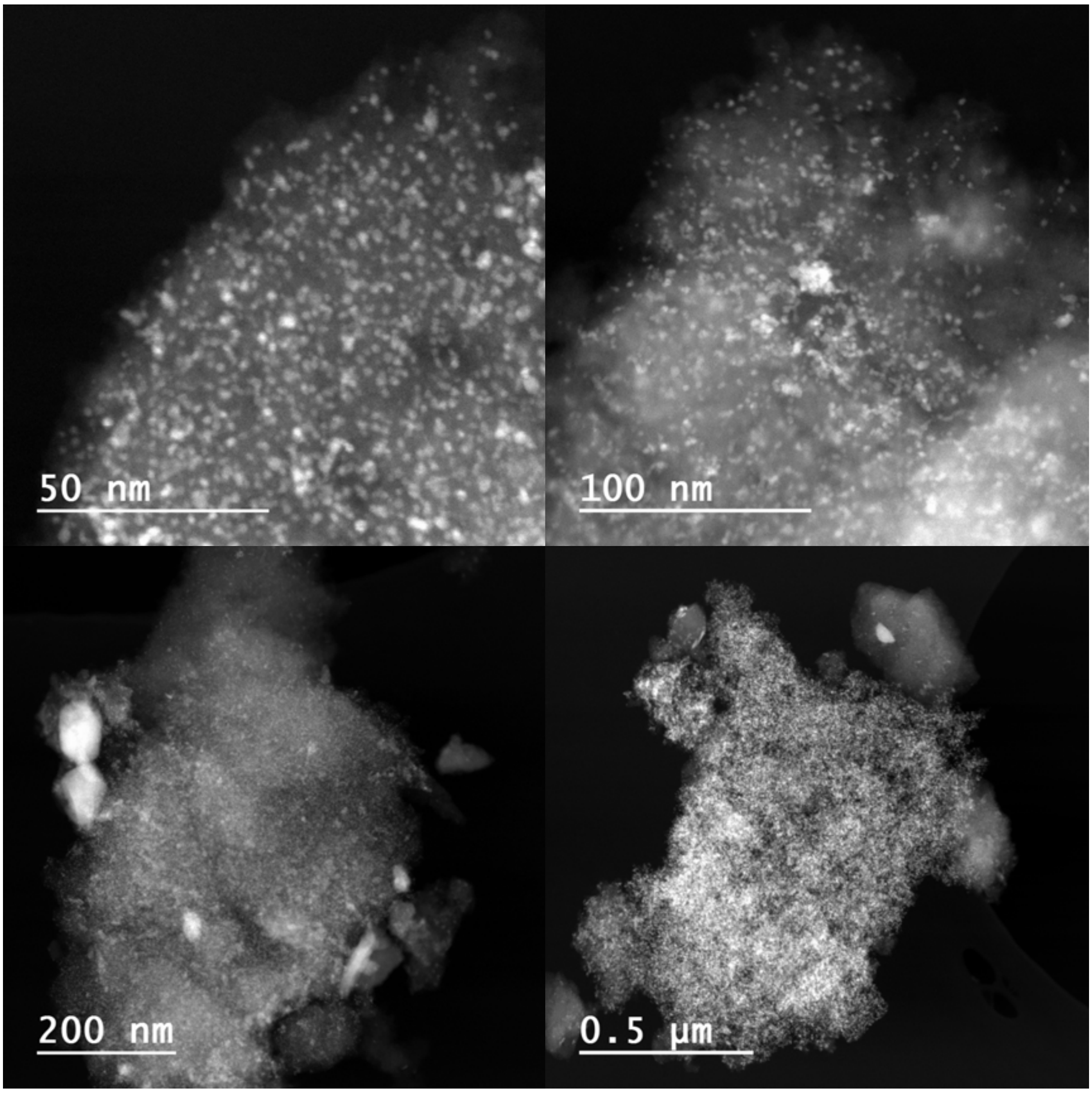

Figure S1. HAADF-STEM images taken at different magnifications showing the typical morphology of the unused Pd/C. Scale bars are given in the bottom left corners of each image.

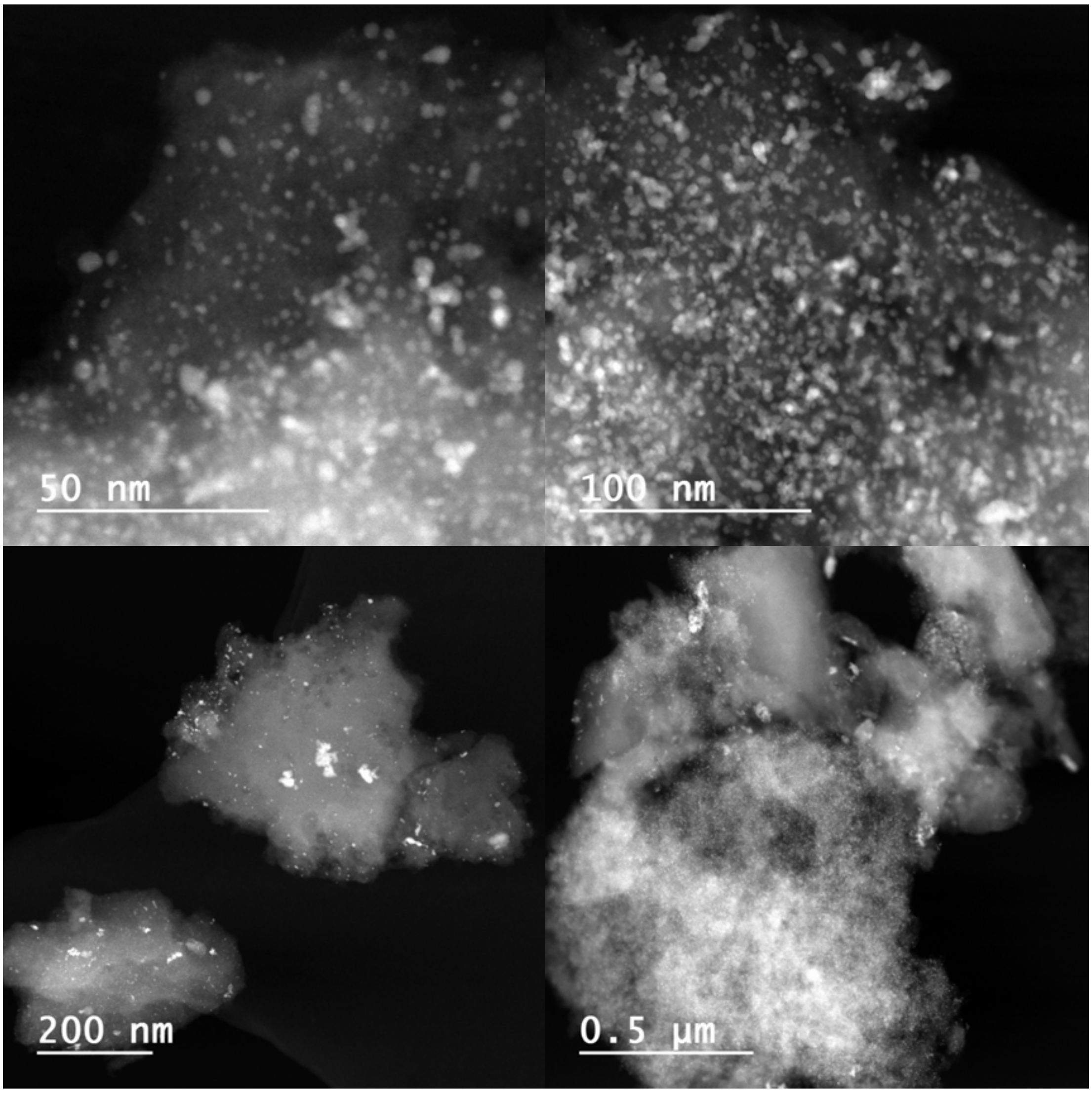

Figure S2. HAADF-STEM images taken at different magnifications showing the typical morphology of the Pd/C after one reaction. Scale bars are given in the bottom left corners of each image.

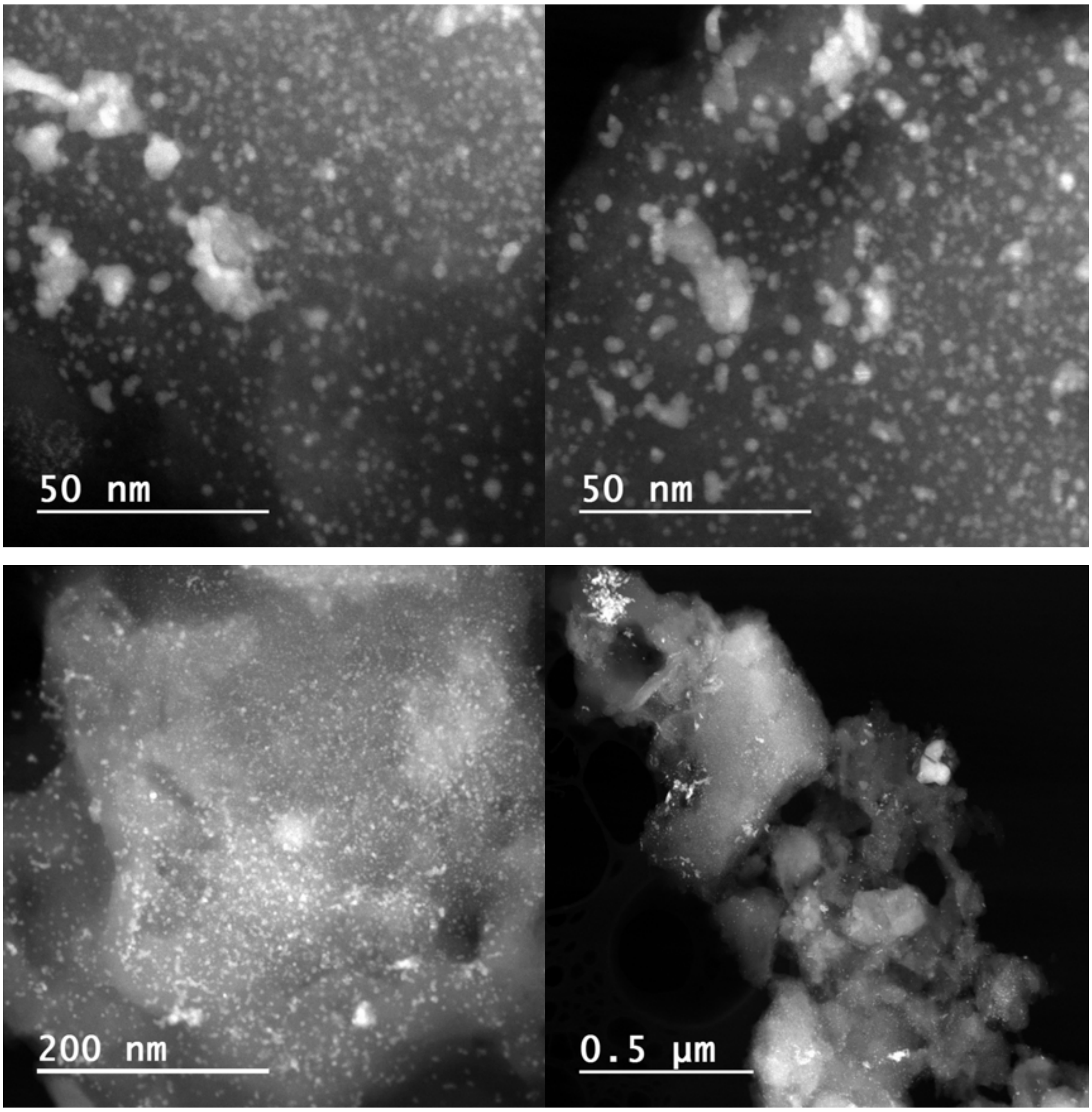

Figure S3. HAADF-STEM images taken at different magnifications showing the typical morphology of the Pd/C after six reactions. Scale bars are given in the bottom left corners of each image.

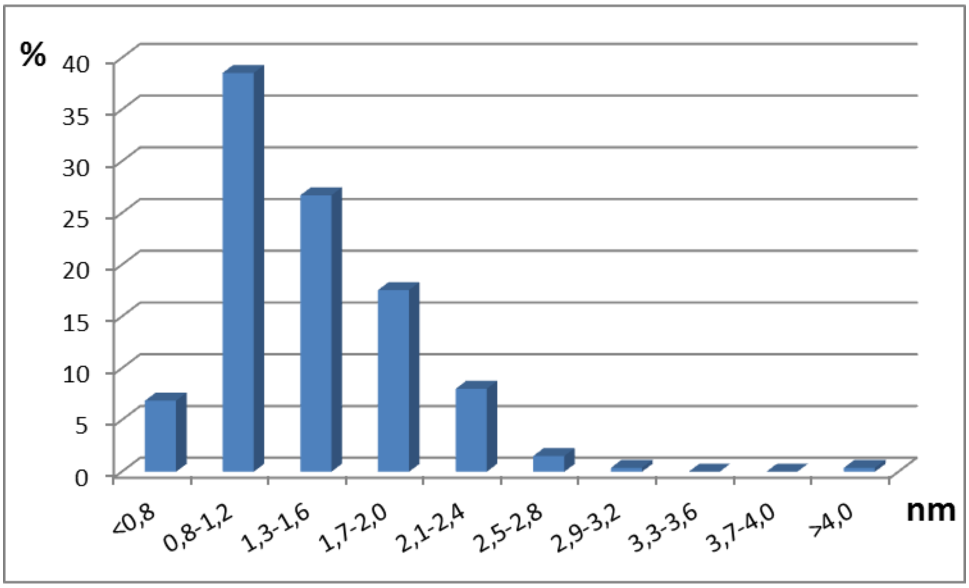

Figure S4: Size distribution of Pd particles in the sample of unused Pd/C, with an average size of 1.43 nm.

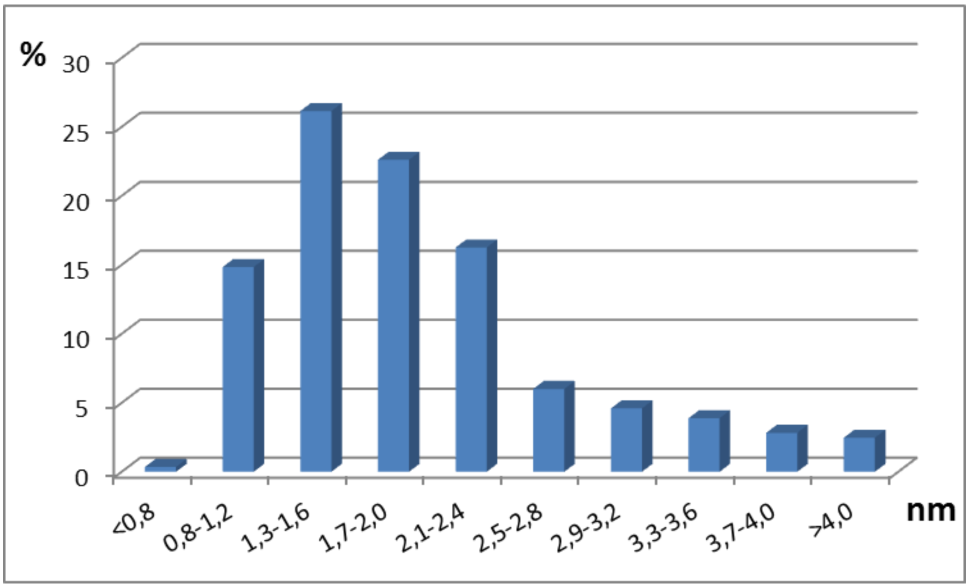

Figure S5: Size distribution of Pd particles in the sample of Pd/C after one reaction, with an average size of 2.02 nm.

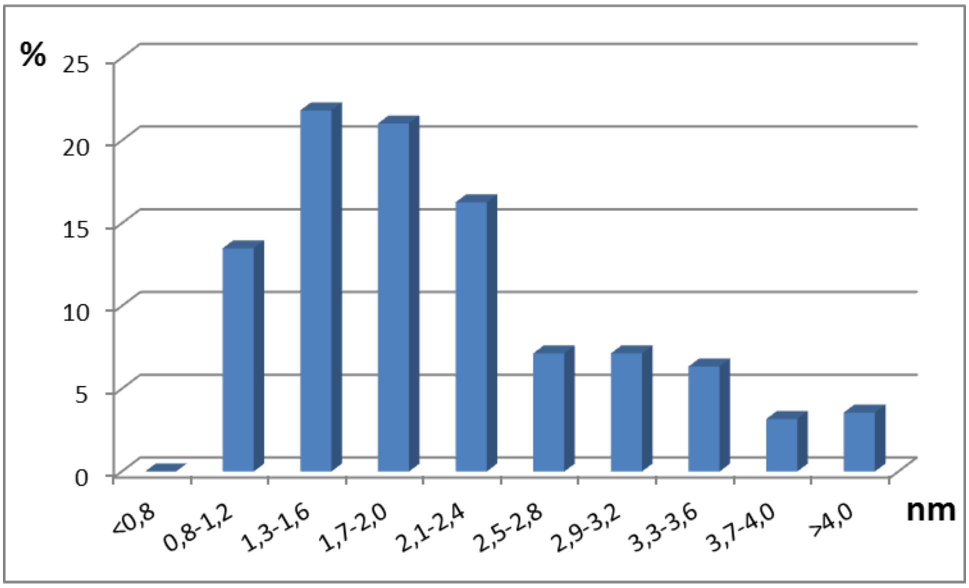

Figure S6: Size distribution of Pd particles in the sample of Pd/C after six reactions, with an average size of 2.24 nm.

Characterization of unused and recycled Pd/C catalyst by XPS

To determine if the Pd particles of the catalyst underwent a change of oxidation state under the employed catalytic conditions, samples of unused and recycled Pd/C were studied by XPS. The values were found to deviate compared to reference bulk values for palladium. However, it is know that the surface concentration of palladium on carbon support has a significant influence on the observed binding energies in XPS analysis. According to the study of Bastl *et al.* the binding energy for Pd(0) could range from 335.1 (for bulk Pd) to above 336 eV (for low surface concentrations) for palladium supported on carbon.[1] Based on these results we could compare the XPS spectral data for the recovered Pd catalyst (Figure S8) to that of an unused catalyst (Figure S7). A clear increase of the relative intensity for the peak belonging to Pd(0) at 335.4 eV together with a decrease of the Pd(II)-peak at 336.8 eV were observed (Figure S8). As depicted in Figure S7, it was estimated that in the unused Pd catalyst, Pd(0) only constitute ~1/3 of the total Pd signal, while in the recovered catalyst it was estimated to ~90% of the total Pd signal (Figure S8).

Peak	Type	Position BE (eV)	FWHM (eV)	Raw Area (cps eV)	RSF	Atomic Mass	Atomic Conc. %	Mass Conc. %
Pd (0)	Comp	335.410	1.600	2008.8	5.356	106.534	33.34	33.34
Pd (II)	Comp	336.796	1.600	4019.4	5.356	106.534	66.66	66.66

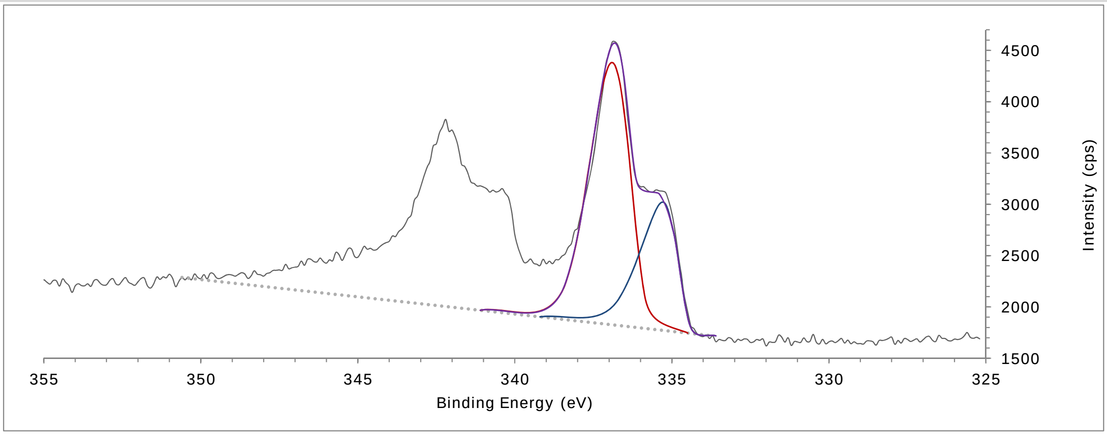

Figure S7. XPS spectra of the unused Pd catalyst. (—) combined Pd peak, (—) Pd(II) component, (—) Pd(0) component.

Peak	Type	Position BE (eV)	FWHM (eV)	Raw Area (cps eV)	RSF	Atomic Mass	Atomic Conc. %	Mass Conc. %
Pd (0)	Comp	335.349	1.400	10116.2	5.356	106.534	92.36	92.36
Pd (II)	Comp	336.758	1.400	837.4	5.356	106.534	7.64	7.64

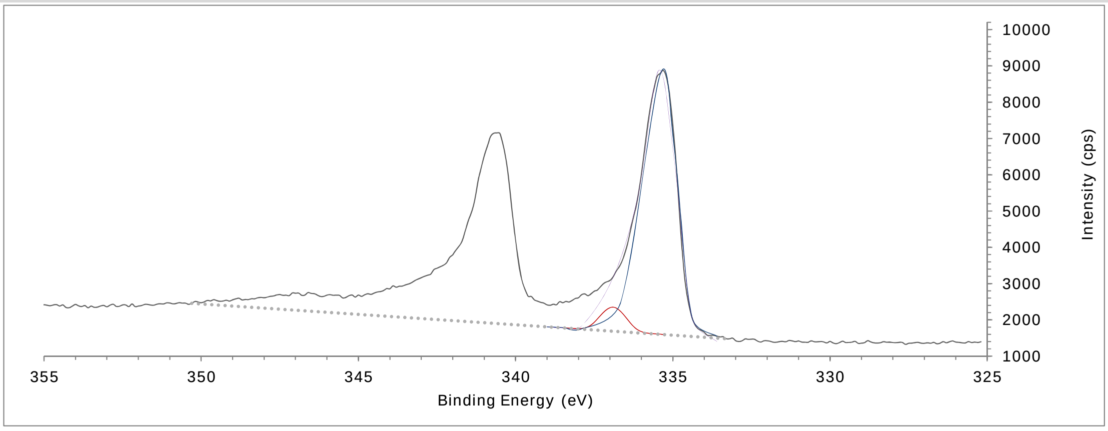

Figure S8. XPS spectra of the recovered Pd catalyst. (—) combined Pd peak, (—) Pd(II) component, (—) Pd(0) component.

Recycling study of the Pd/C in the deoxygenation reaction

4-Methoxyacetophenone (0.5 mmol, 75 mg) and Pd/C (4.23 wt%, 5.3 mg, 0.0021 mmol Pd) were suspended in MeOH (1.5 mL) in a sealed microwave tube. To this was added chlorobenzene (5 μL, 0.025 mmol) and PMHS (0.09 mL, 3 equiv of [H-]) under stirring. The reaction mixture was then allowed to stir at rt for 2 h. The catalyst was separated by centrifugation and the supernatant was collected. The catalyst was washed with MeOH (3 × 4 mL) and the organic layers were collected, combined with the supernatant and concentrated in vacuo. The conversion of the reaction was determined from analysis by ^{1}H-NMR. This procedure was repeated six times, after which the Pd nanocatalyst was collected and analyzed by TEM.

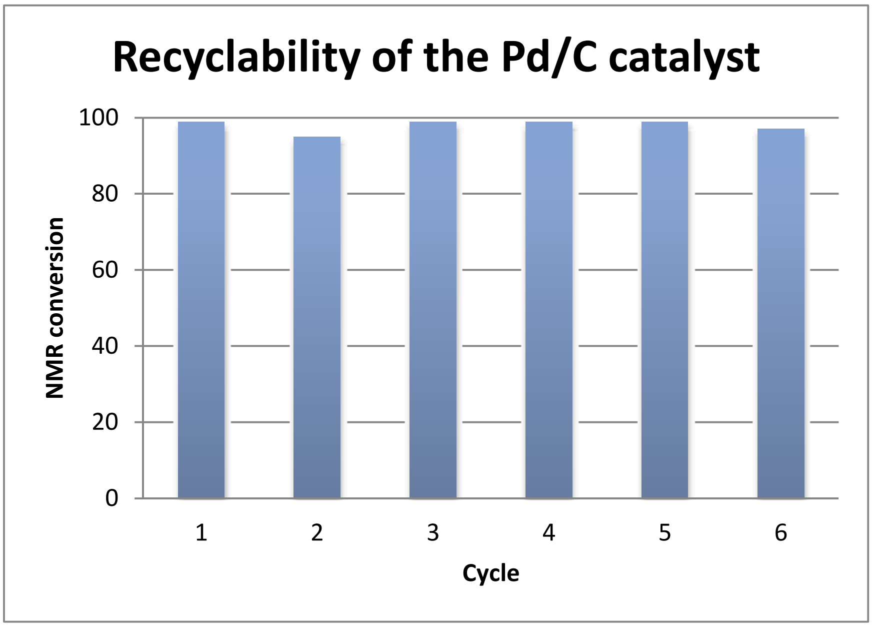

Figure S9. Recyclability of the Pd/C catalyst over six cycles under the optimized reaction conditions.

Compound characterization.

Not isolated products:

Toluene

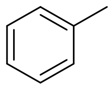

NMR yield: >95%. Spectral data was in accordance with previously published data.[2]

1-Methyl-4-(trifluoromethyl)benzene

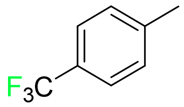

NMR yield: >95%. Spectral data was in accordance with previously published data.[3]

Ethylbenzene

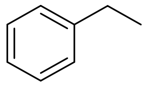

NMR yield: >95%. Spectral data was in accordance with previously published data.[4]

4-Ethylanisole

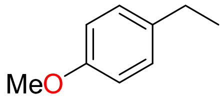

NMR yield: >95%. Spectral data was in accordance with previously published data.[5]

1-Ethyl-4-fluorobenzene

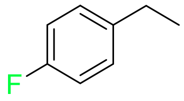

NMR yield: >95%. Spectral data was in accordance with previously published data.[6]

1-Ethyl-4-(trifluoromethyl)benzene

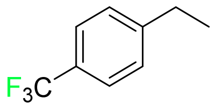

NMR yield: >95%. Spectral data was in accordance with previously published data. [7]

Methyl 4-ethylbenzoate

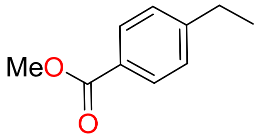

NMR yield: >95%.Spectral data was in accordance with previously published data.[8]

4-Ethylaniline

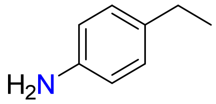

NMR yield: >95%. Spectral data was in accordance with previously published data.[8]

1-Ethyl-2,4-dimethylbenzene

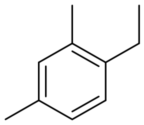

NMR yield: >95%. Spectral data was in accordance with previously published data.[9]

Isolated products:

4-Ethyl-1,1'-biphenyl

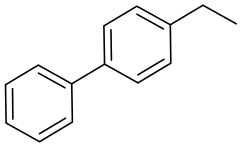

0.091 g, 99% yield; **^{1}H-NMR** (400 MHz, CDCl3): δ = 7.63 – 7.58 (m, 2H), 7.56 – 7.51 (m, 2H), 7.48 – 7.41 (m, 2H), 7.38 – 7.27 (m, 3H), 2.72 (q, *J* = 7.6 Hz, 2H), 1.30 (t, *J* = 7.6 Hz, 3H); **^{13}C-NMR** (100 MHz, CDCl3): δ = 143.5, 141.3, 138.8, 128.4, 127.2, 127.2, 127.1, 28.7, 15.7.

***N*-(4-Ethylphenyl)acetamide**

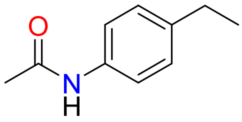

0.073 g, 90% yield; **^{1}H-NMR** (400 MHz, CDCl3): δ = 7.41 – 7.35 (m, 3H), 7.15 – 7.12 (m, 2H), 2.61 (q, *J* = 7.6 Hz, 2H), 2.15 (s, 3H), 1.21 (t, 7.6 Hz, 3H); **^{13}C-NMR** (100 MHz, CDCl3): δ = 168.4, 140.5, 135.6, 128.4, 120.3, 28.4, 24.6, 15.7.

5-Methoxy-2,3-dihydro-1H-indene

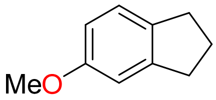

0.071 g, 96% yield; **^{1}H-NMR** (400 MHz, CDCl3): δ = 7.13 – 7.08 (m, 1H), 6.81 – 6.76 (m, 1H), 6.72 – 6.67 (m, 1H), 3.79 (s, 3H), 2.92 – 2.81 (m, 4H), 2.12 – 2.02 (m, 2H) ; **^{13}C-NMR** (100 MHz, CDCl3): δ = 158.7, 145.9, 136.3, 124.9, 112.0, 110.0, 55.6, 33.3, 32.1, 25.9.

(Cyclohexylmethyl)benzene

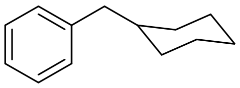

0.076 g, 88% yield; **^{1}H-NMR** (400 MHz, CDCl3): δ = 7.29 – 7.25 (m, 2H), 7.20 – 7.13 (m, 3H), 2.50 – 2.47 (m, 1H), 1.73 – 1.60 (m, 5H), 1.58 – 1.45 (m, 1H), 1.27 – 1.10 (m, 3H), 1.01 – 0.86 (m, 2H); **^{13}C-NMR** (100 MHz, CDCl3): δ = 141.5, 129.3, 128.2, 125.7, 44.3, 39.9, 33.3, 26.7, 26.5.

Diphenylmethane

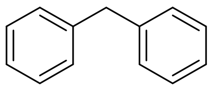

0.084 g, 99% yield; ^{1}**H-NMR** (400 MHz, CDCl3): δ = 7.32 – 7.16 (m, 10H), 3.99 (s, 2H); ^{13}**C-NMR** (100 MHz, CDCl3): δ = 141.3, 129.1, 128.6, 126.2, 42.1.

5-Ethyl-2,3-dihydrobenzofuran

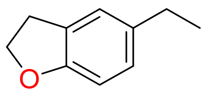

0.065 g, 87% yield; ^{1}**H-NMR** (400 MHz, CDCl3): δ = 7.05 – 7.03 (m, 1H), 6.96 – 6.92 (m, 1H), 6.72 – 6.68 (m, 1H), 4.54 (t, *J* = 8.7 Hz, 2H), 3.18 (t, *J* = 8.7 Hz, 2H), 2.58 (q, *J* = 7.6 Hz, 2H), 1.21 (t, *J* = 7.6 Hz, 3H); ^{13}**C-NMR** (100 MHz, CDCl3): δ = 158.2, 136.5, 127.3, 127.0, 124.5, 109.0, 71.3, 30.0, 28.5, 16.4.

2-Ethoxy-4-methylphenol

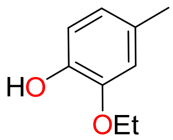

0.070 g, 92% yield; ^{1}**H-NMR** (400 MHz, CDCl3): δ = 6.85 – 6.80 (m, 1H), 6.77 – 6.65 (m, 2H), 5.53 (s, 1H), 4.10 (q, *J* = 7.0 Hz, 2H), 2.29 (s, 3H); 1.44 (t, *J* = 7.0 Hz, 3H); ^{13}**C-NMR** (100 MHz, CDCl3): δ = 145.7, 143.6, 129.6, 121.6, 114.2, 112.7, 64.5, 21.2, 15.1.

2-Phenylacetic acid

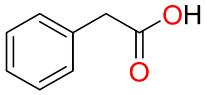

0.068 g, 99% yield; ^{1}**H-NMR** (400 MHz, CDCl3): δ = 10.79 (bs, 1H), 7.38 – 7.27 (m, 5H), 3.66 (s, 2H); ^{13}**C-NMR** (100 MHz, CDCl3): δ = 178.2, 133.4, 129.5, 128.8, 127.5, 41.2.

2-Propylphenol

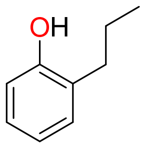

0.063 g, 93% yield; ^{1}**H-NMR** (400 MHz, CDCl3): δ = 7.17 – 7.07 (m, 2H), 6.93 – 6.87 (m ,1H), 6.80 – 6.74 (m, 1H), 2.64 – 2.58 (m, 2H), 1.73 – 1.62 (m, 2H), 1.04 – 1.97 (m, 3H); ^{13}**C-NMR** (100 MHz, CDCl3): δ = 153.5, 130.4, 128.5, 128.5, 127.2, 120.9, 115.3, 32.1, 23.0, 14.1.

Ethylferrocene

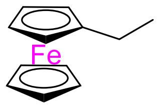

0.103 g, 96% yield; ^{1}**H-NMR** (400 MHz, CDCl3): δ = 4.11 (s, 5H), 4.09 – 4.07 (m, 2H), 4.06 – 4.04 (m, 2H), 2.35 (q, *J* = 7.5 Hz, 2H), 1.18 (t, *J* = 7.5 Hz, 3H); ^{13}**C-NMR** (100 MHz, CDCl3): δ = 91.3, 68.5, 67.6, 67.1, 22.4, 14.8.

4-Phenylbutan-2-one

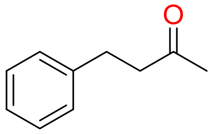

0.059 g, 80% yield; [1]**H-NMR** (400 MHz, CDCl3): δ = 7.31 – 7.26 (m, 2H), 7.22 – 7.17 (m, 3H), 2.93 – 2.87 (m, 2H), 2.79 – 2.74 (m, 2H), 2.14 (s, 3H); [13]**C-NMR** (100 MHz, CDCl3): δ = 207.9, 141.1, 128.6, 128.4, 126.2, 45.3, 30.1, 29.8.

Reference

1. Z. Bastl, O. Přibyl, P. Mikušík, *Czech. J. Phys. B* **1984**, *34*, 981-988

2. L. Zhou; L.-Z. Wu; L.-P Zhang.; C.-H.Tung, *Organometallics* **2006**, 25, 1707-1711

3. Y. Ye; S. A. Künzi;M. S. Sanford, *Org. Lett.* **2012**, 14, 4979–4981

4. N.Sakai; T. Moriya; T. Konakahara*, J. Org. Chem*. **2007**, *72*, 5920–5922

5. M. Tomohiro; T. Tohru; Y. Masatoshi; S. Hiroyasu; M. Yasunari; S. Hironao, *Adv. Synth. Catal.* **2009**, *351*, 2091–209

6. T. S. Carter; L. Guiet; D. J. Frank; J. West;S. P. Thomas, *Adv. Synth. Catal .* **2013**, *355*, 880–884

7. P. J. Rushworth; D. G. Hulcoop; D. J. Fox*, J. Org. Chem.* **2013**, *78*, 9517–9521

8. M. Huang, L. Wang;X. Zhu, Z. Mao, D. Kuang, Y. Wan, *Eur. J. Org. Chem.* **2012**, *26*, 4897 - 4901

9. SDBSWeb : http://sdbs.db.aist.go.jp (National Institute of Advanced Industrial Science and Technology, date of access: 2014-10-13)

Spectroscopic data.

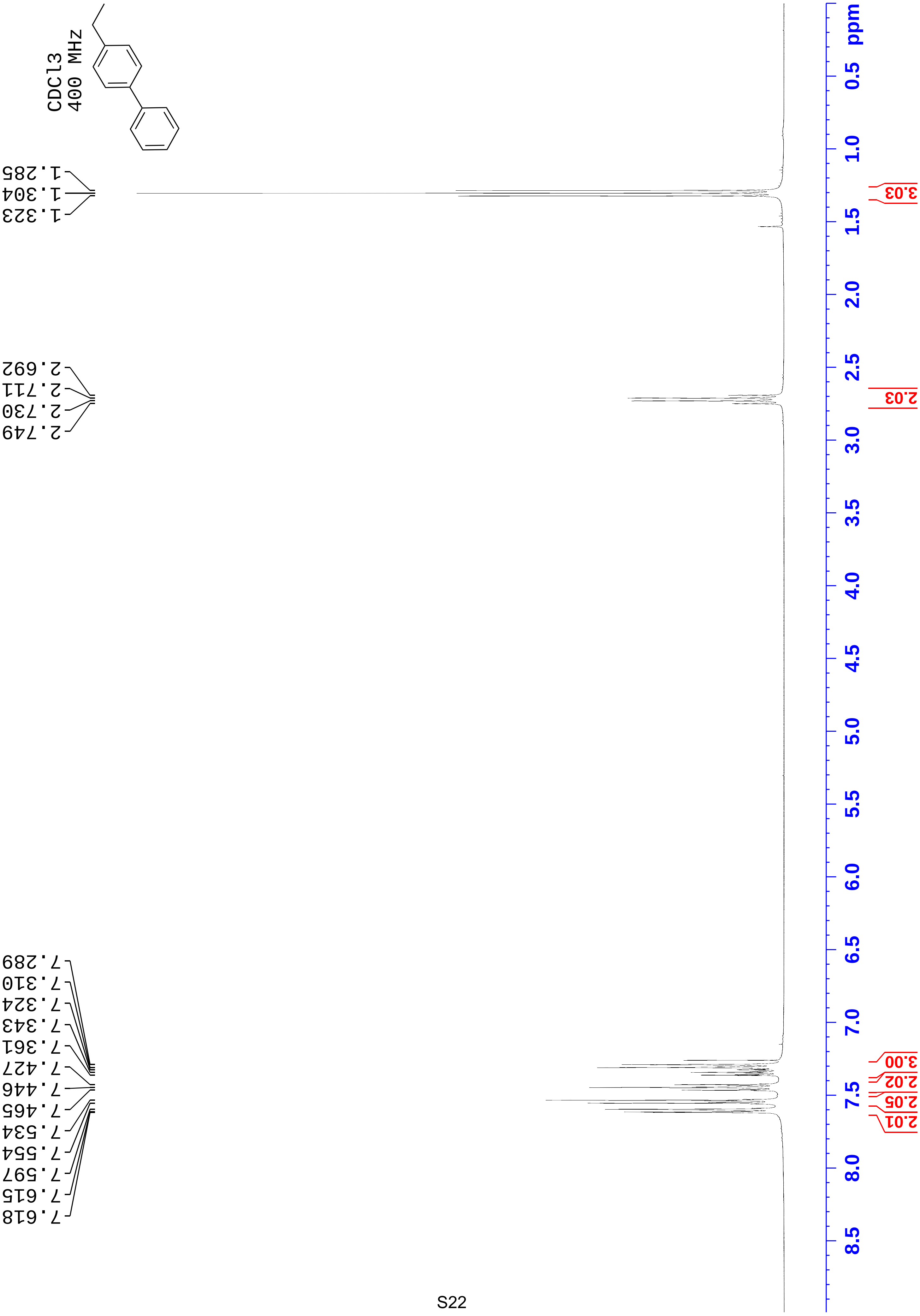
CDCl3
400 MHz
1.285
1.304
1.323
2.692
2.711
2.730
2.749
7.289
7.310
7.324
7.343
7.361
7.427
7.446
7.465
7.534
7.554
7.597
7.615
7.618
3.03
2.03
3.00
2.02
2.05
2.01
ppm

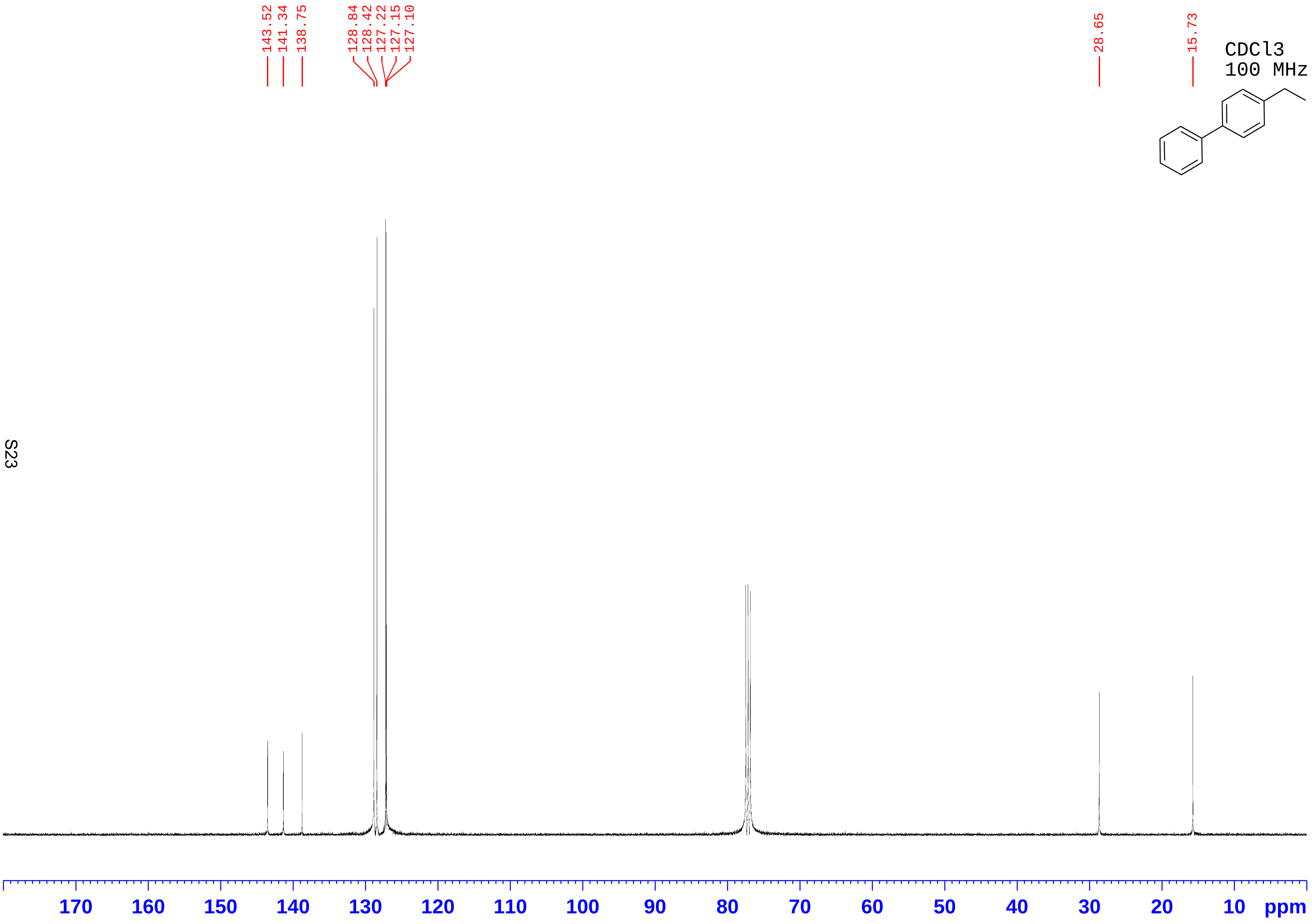
CDCl3
100 MHz
143.52
141.34
138.75
128.84
128.42
127.22
127.15
127.10
28.65
15.73
170
160
150
140
130
120
110
100
90
80
70
60
50
40
30
20
10
ppm

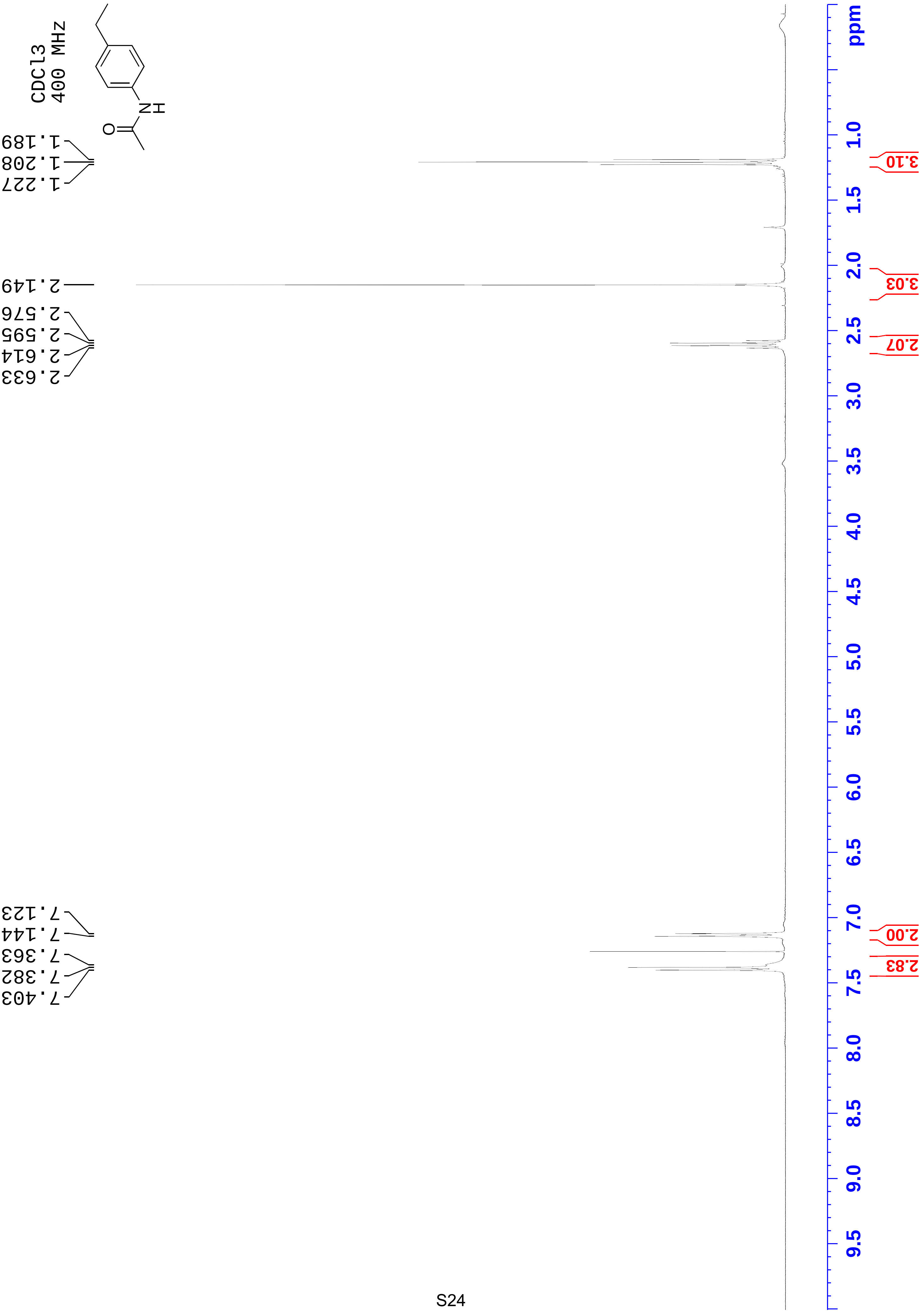
CDCl3
400 MHz
1.189
1.208
1.227
2.149
2.576
2.595
2.614
2.633
7.123
7.144
7.363
7.382
7.403
3.10
3.03
2.07
2.00
2.83
ppm

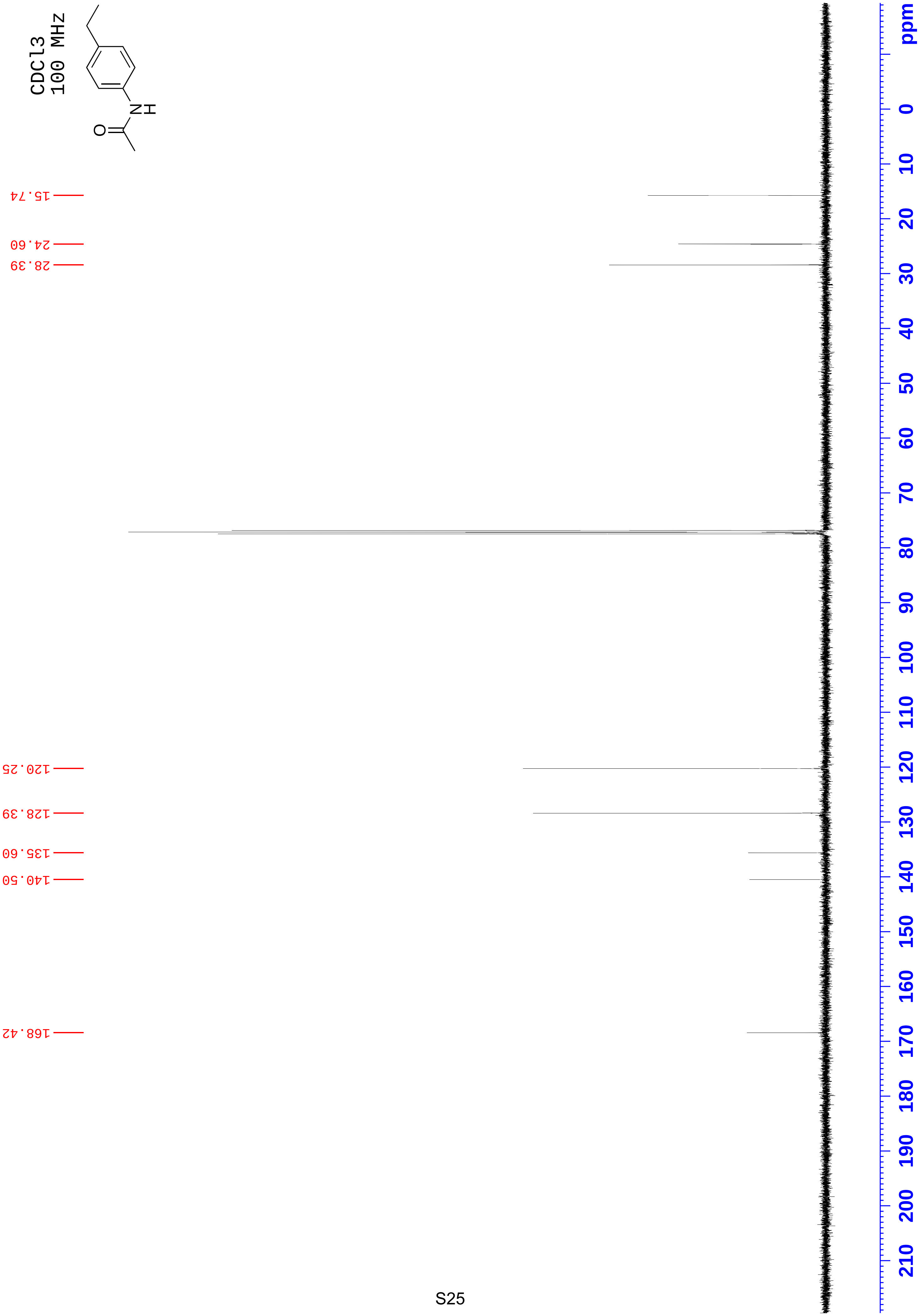
CDCl3
100 MHz
O
N
H
15.74
24.60
28.39
120.25
128.39
135.60
140.50
168.42
ppm
0
10
20
30
40
50
60
70
80
90
100
110
120
130
140
150
160
170
180
190
200
210

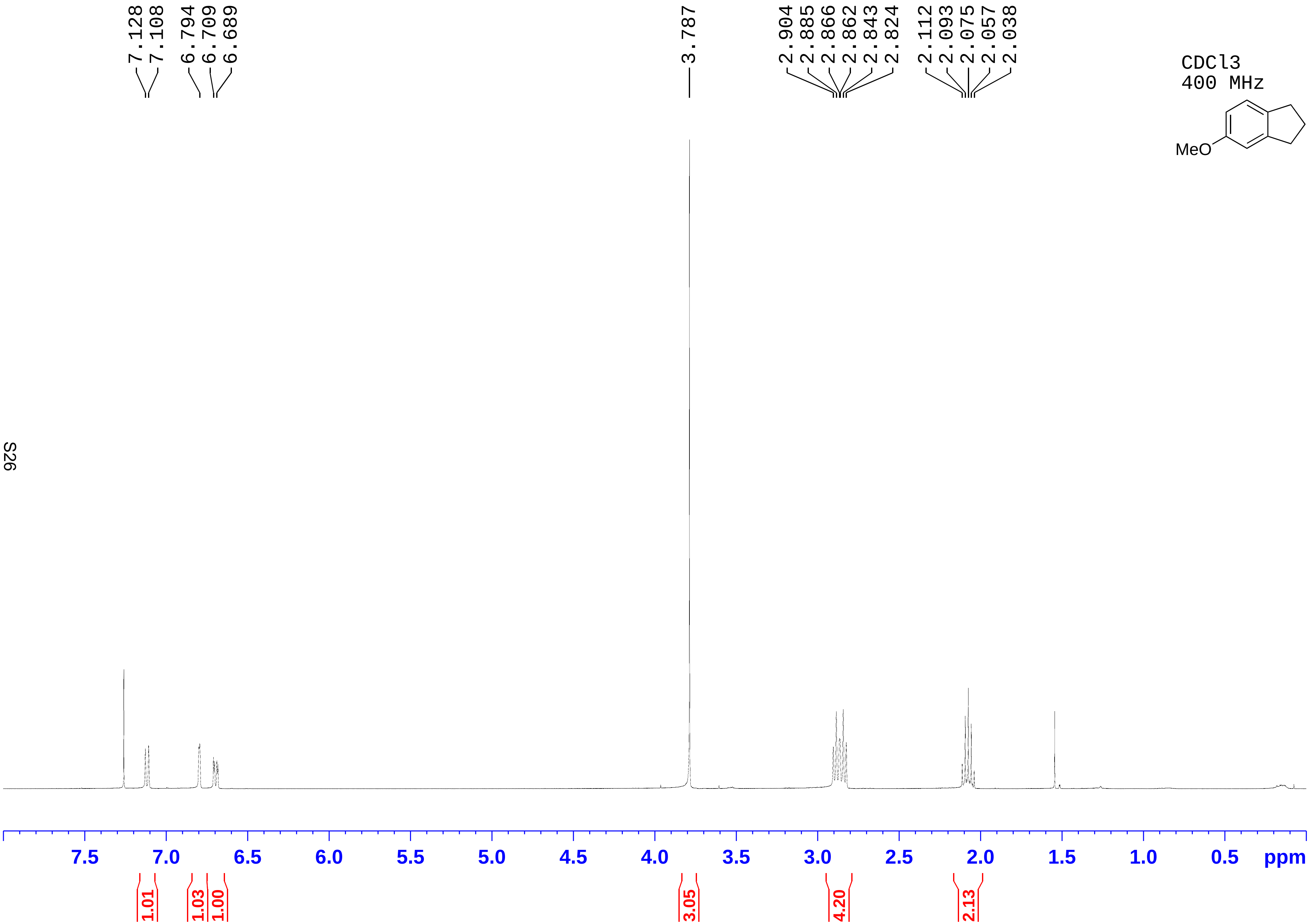
7.128
7.108
6.794
6.709
6.689
3.787
2.904
2.885
2.866
2.862
2.843
2.824
2.112
2.093
2.075
2.057
2.038
CDCl3
400 MHz
MeO
7.5
7.0
6.5
6.0
5.5
5.0
4.5
4.0
3.5
3.0
2.5
2.0
1.5
1.0
0.5
ppm
1.01
1.03
1.00
3.05
4.20
2.13

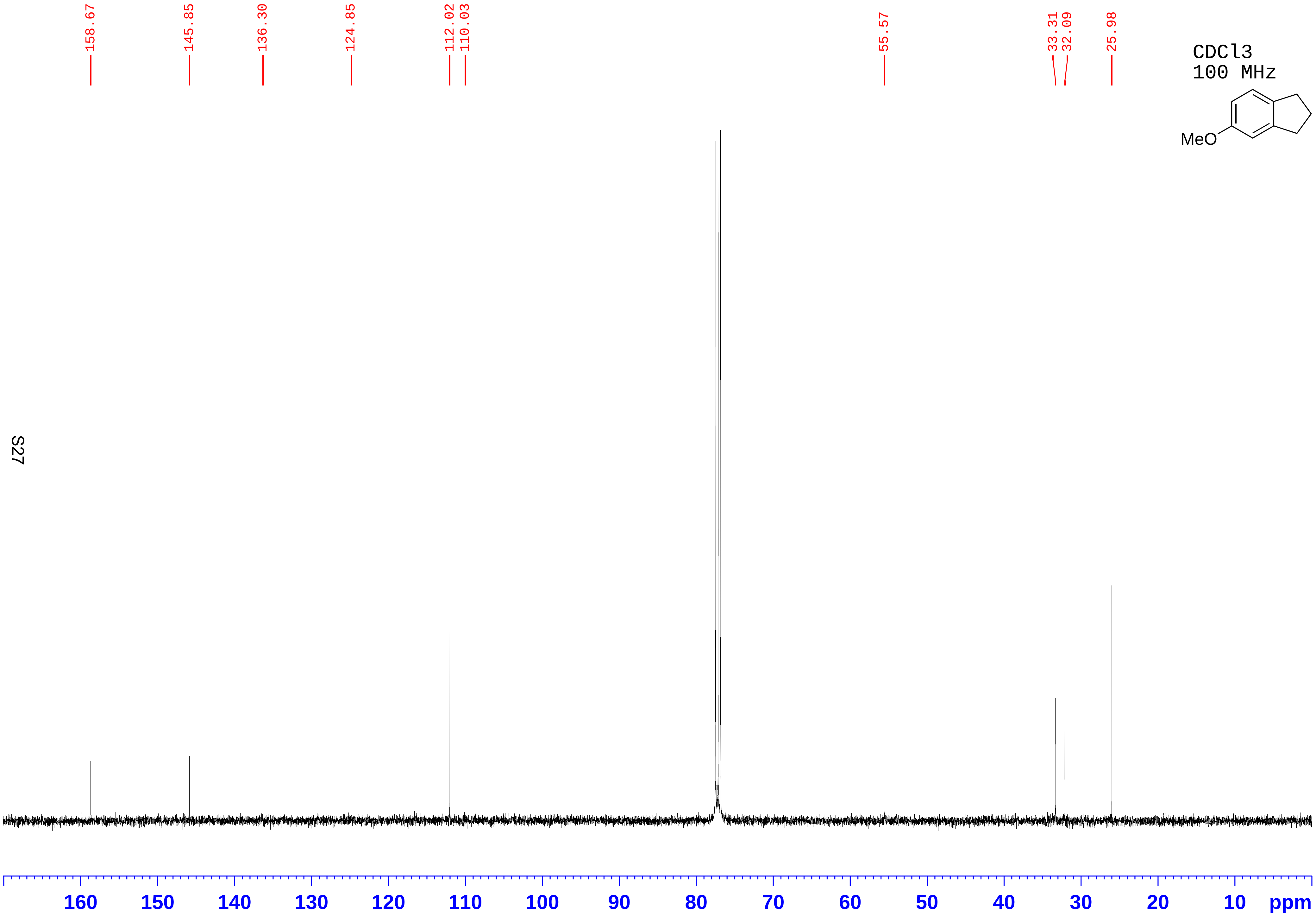
158.67
145.85
136.30
124.85
112.02
110.03
55.57
33.31
32.09
25.98
CDCl3
100 MHz
MeO
160
150
140
130
120
110
100
90
80
70
60
50
40
30
20
10
ppm

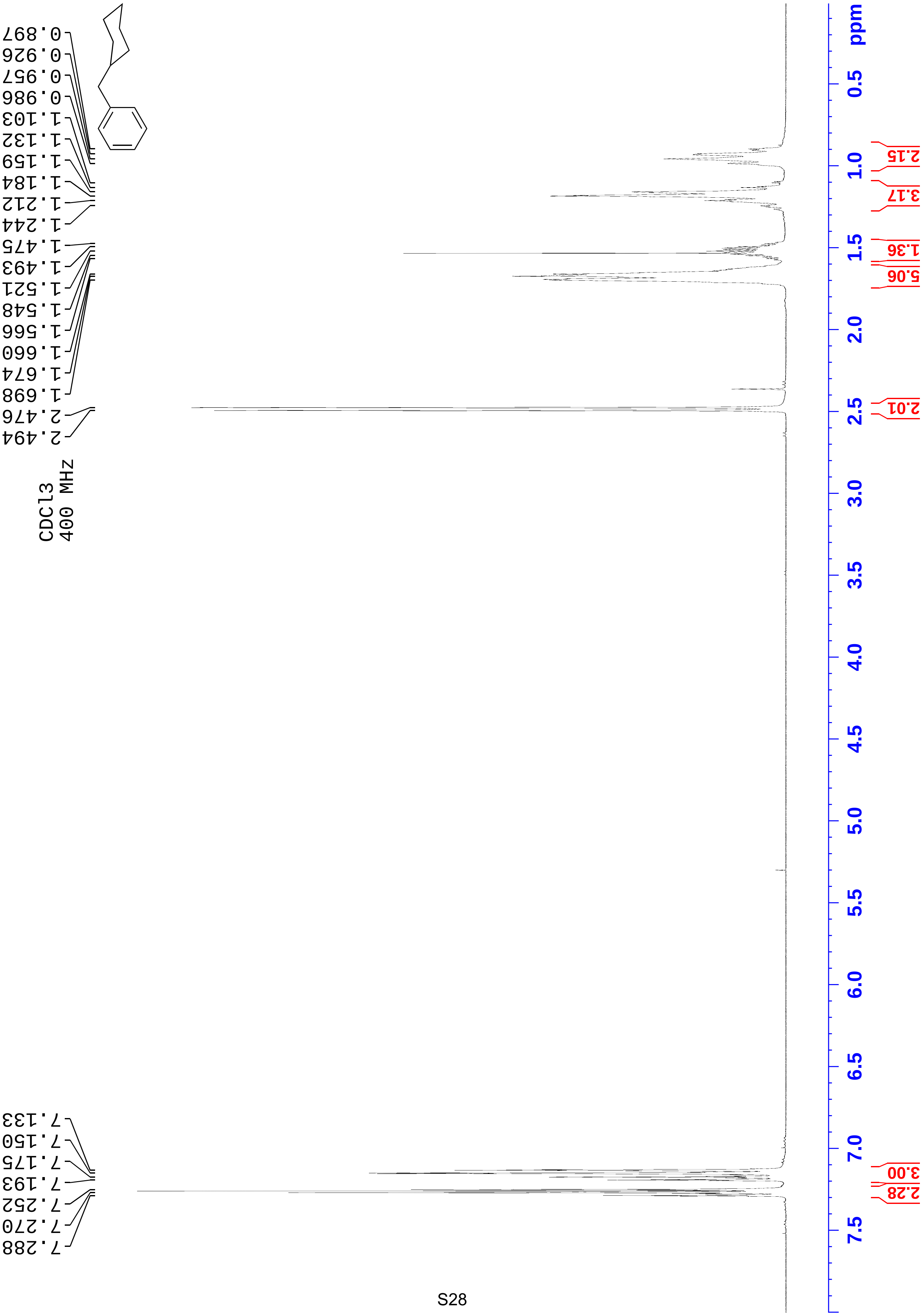
0.897
0.926
0.957
0.986
1.103
1.132
1.159
1.184
1.212
1.244
1.475
1.493
1.521
1.548
1.566
1.660
1.674
1.698
2.476
2.494
CDCl3
400 MHz
7.133
7.150
7.175
7.193
7.252
7.270
7.288
2.15
3.17
1.36
5.06
2.01
3.00
2.28
ppm
0.5
1.0
1.5
2.0
2.5
3.0
3.5
4.0
4.5
5.0
5.5
6.0
6.5
7.0
7.5

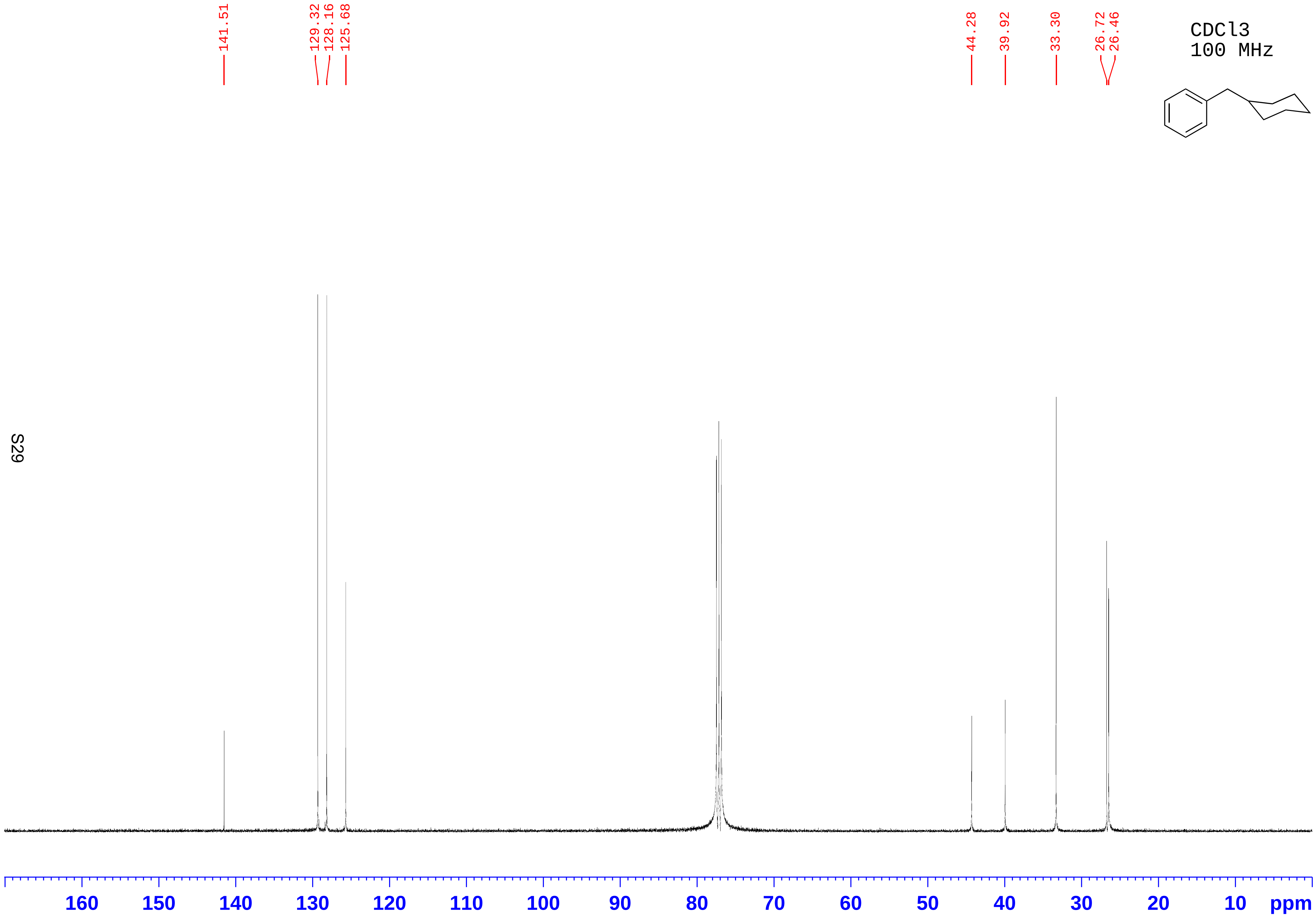

CDCl3
100 MHz
141.51
129.32
128.16
125.68
44.28
39.92
33.30
26.72
26.46
160 150 140 130 120 110 100 90 80 70 60 50 40 30 20 10 ppm

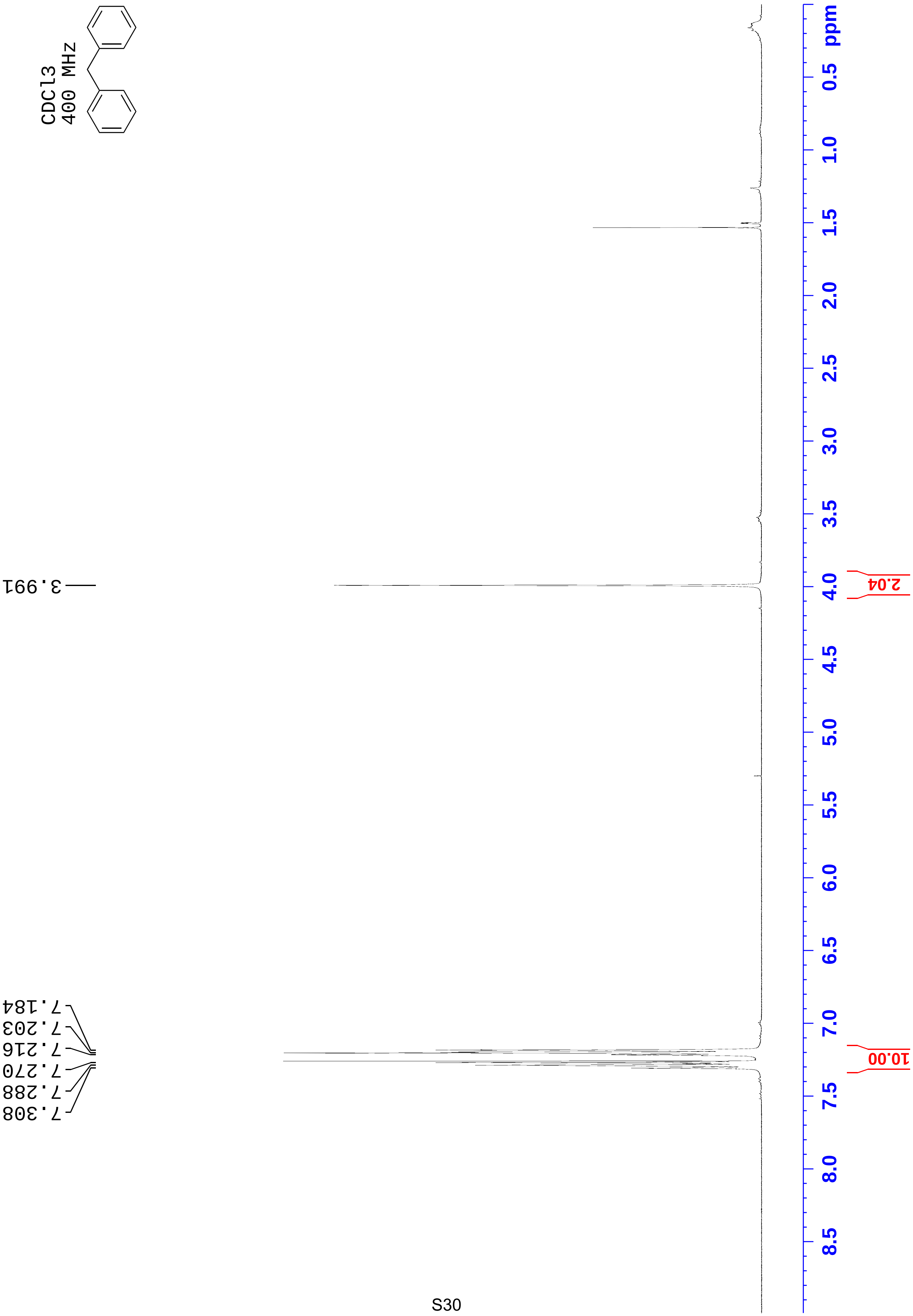

CDCl3
400 MHz
3.991
7.184
7.203
7.216
7.270
7.288
7.308
2.04
10.00
ppm
0.5
1.0
1.5
2.0
2.5
3.0
3.5
4.0
4.5
5.0
5.5
6.0
6.5
7.0
7.5
8.0
8.5

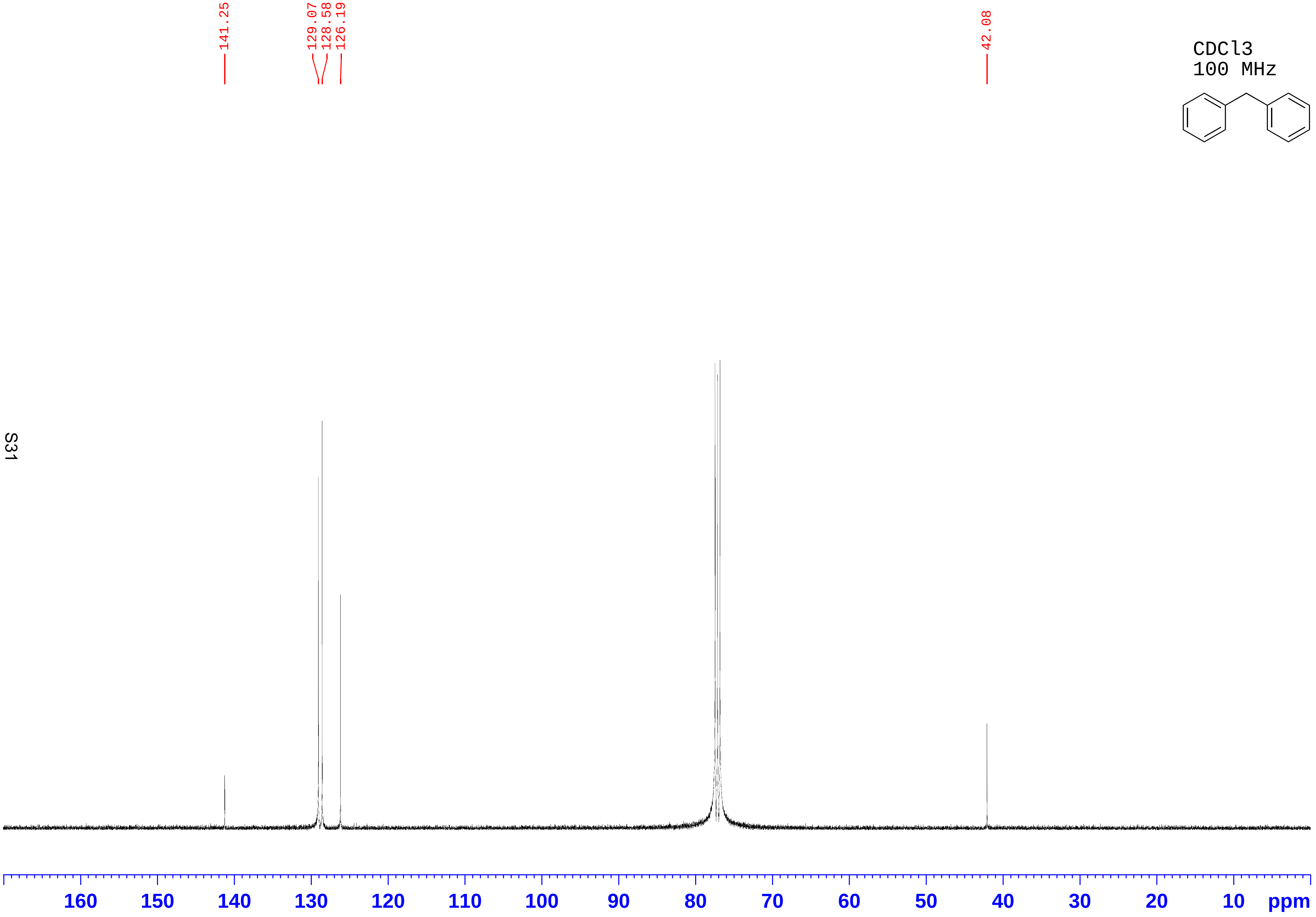
141.25
129.07
128.58
126.19
42.08
CDCl3
100 MHz
160
150
140
130
120
110
100
90
80
70
60
50
40
30
20
10
ppm

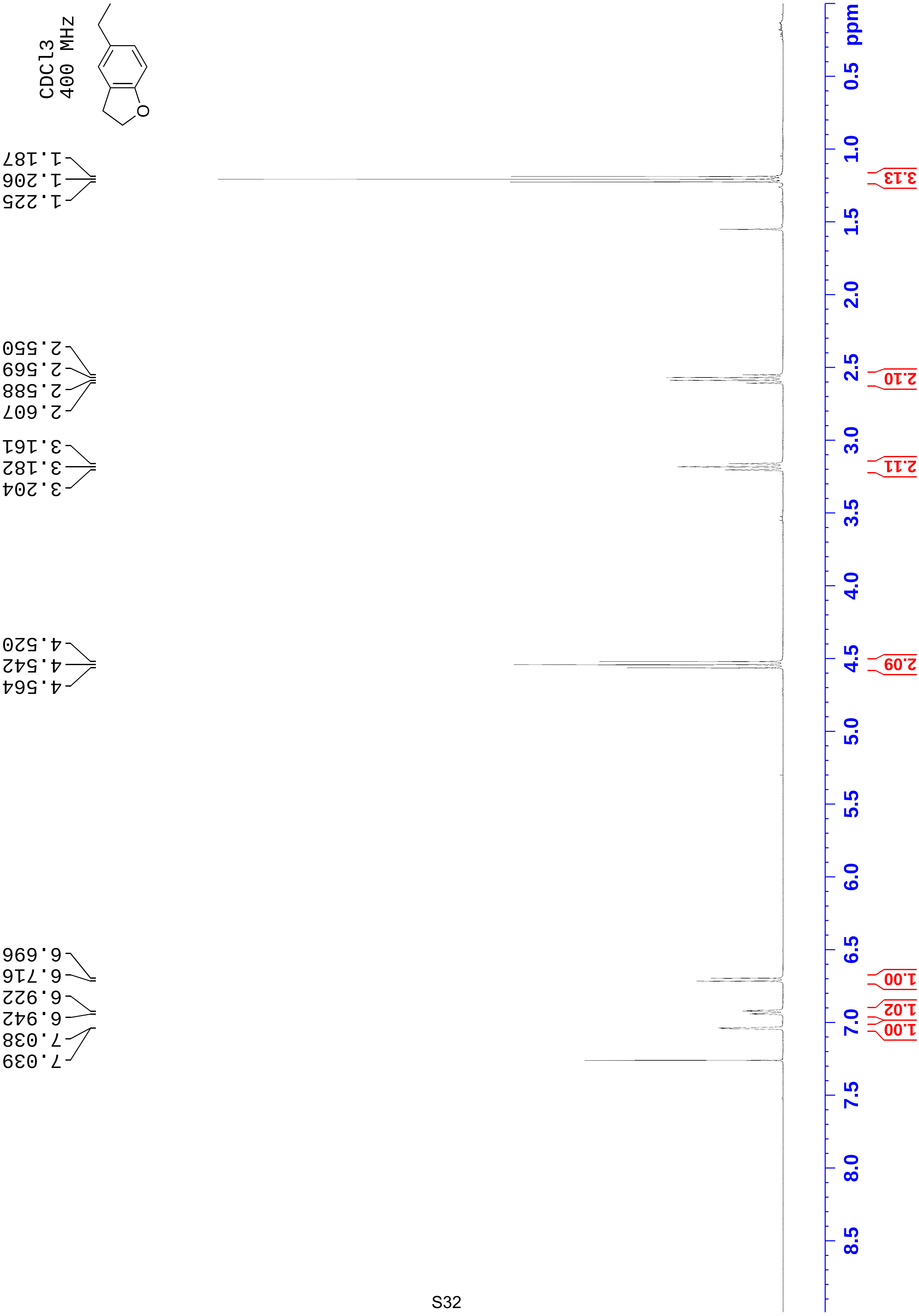
CDCl3
400 MHz
O
1.187
1.206
1.225
2.550
2.569
2.588
2.607
3.161
3.182
3.204
4.520
4.542
4.564
6.696
6.716
6.922
6.942
7.038
7.039
ppm
0.5
1.0
1.5
2.0
2.5
3.0
3.5
4.0
4.5
5.0
5.5
6.0
6.5
7.0
7.5
8.0
8.5
3.13
2.10
2.11
2.09
1.00
1.02
1.00

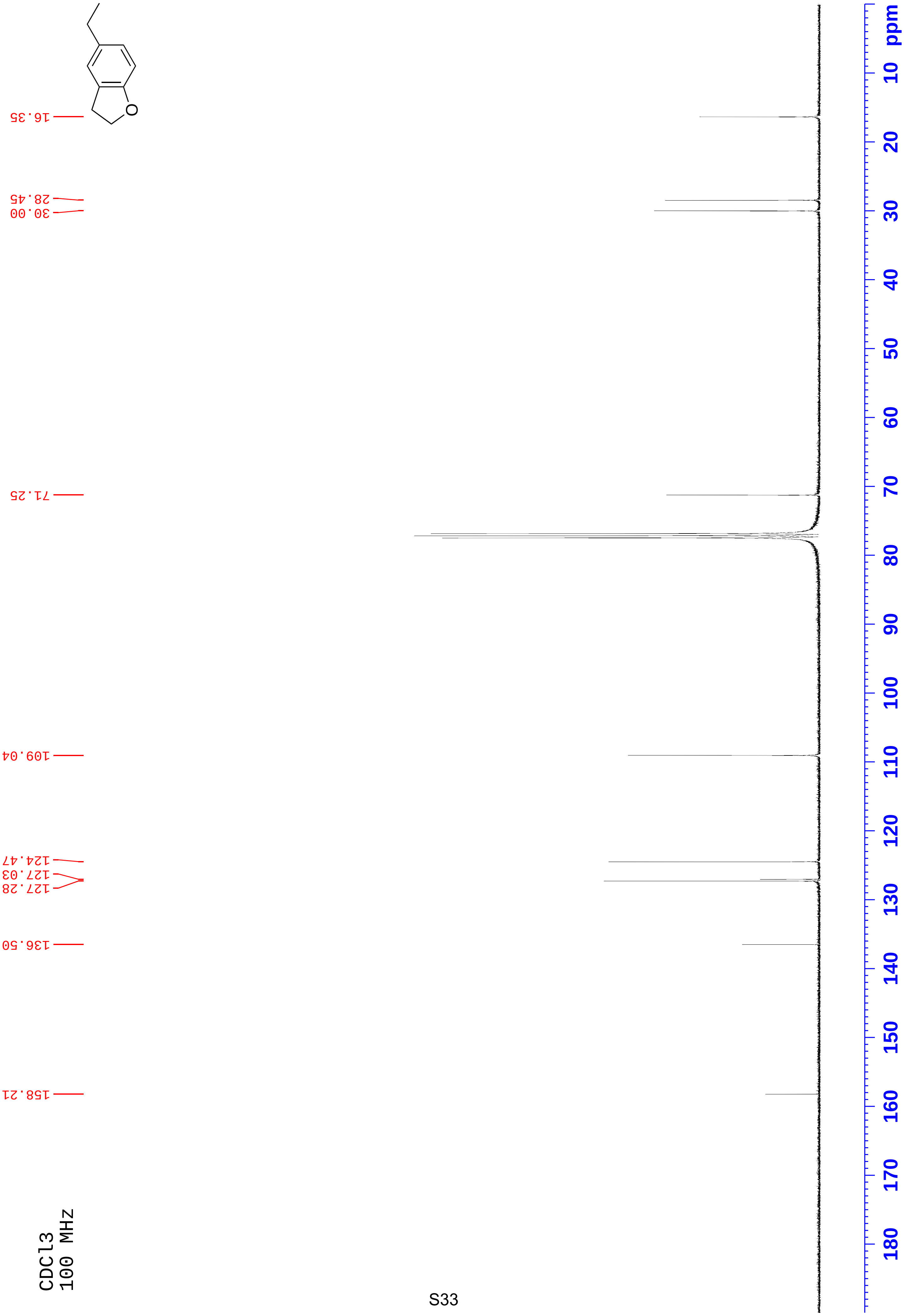
16.35
28.45
30.00
71.25
109.04
124.47
127.03
127.28
136.50
158.21
CDCl3
100 MHz
ppm

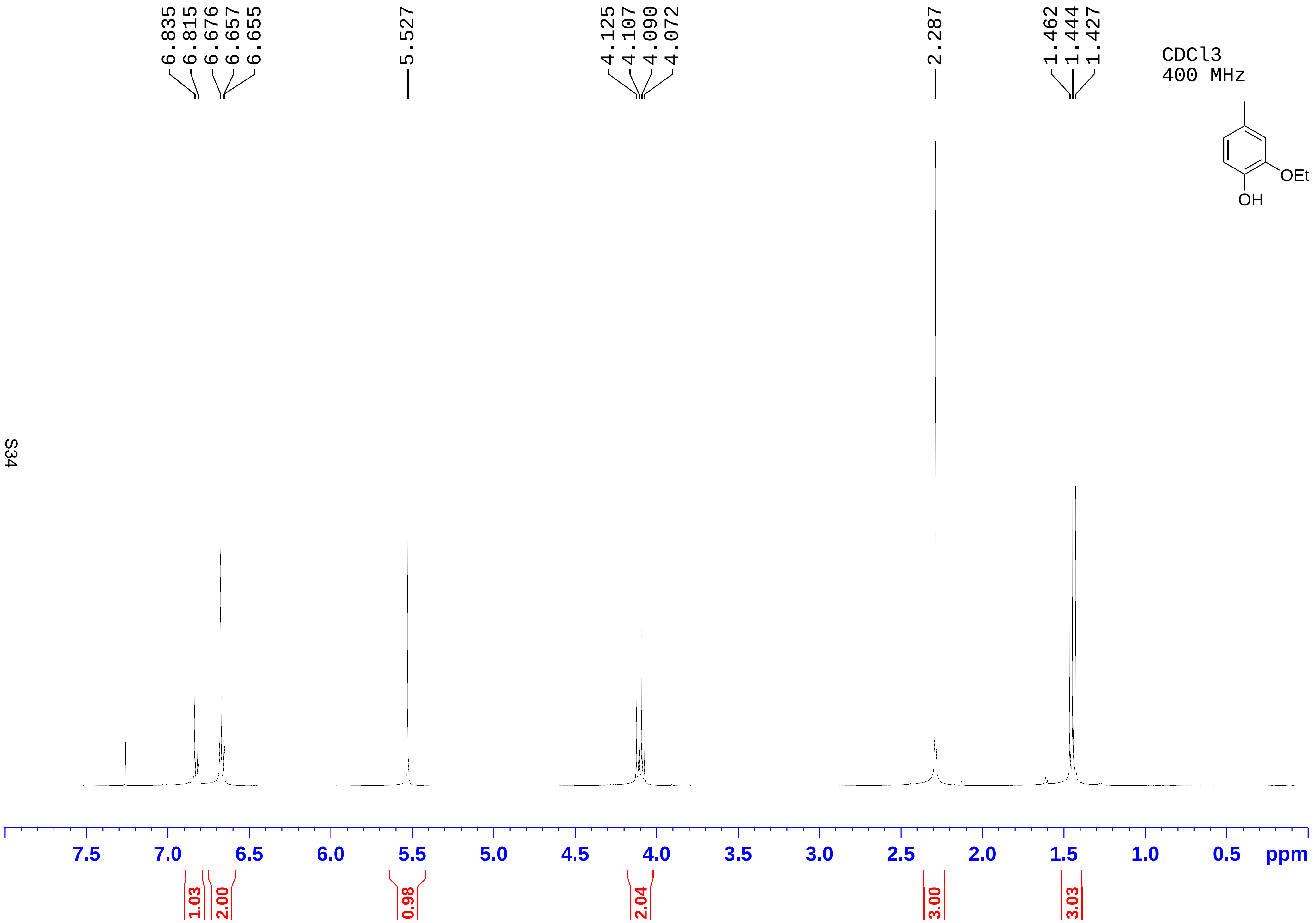
6.835
6.815
6.676
6.657
6.655
5.527
4.125
4.107
4.090
4.072
2.287
1.462
1.444
1.427
CDCl3
400 MHz
OEt
OH
7.5
7.0
6.5
6.0
5.5
5.0
4.5
4.0
3.5
3.0
2.5
2.0
1.5
1.0
0.5
ppm
1.03
2.00
0.98
2.04
3.00
3.03

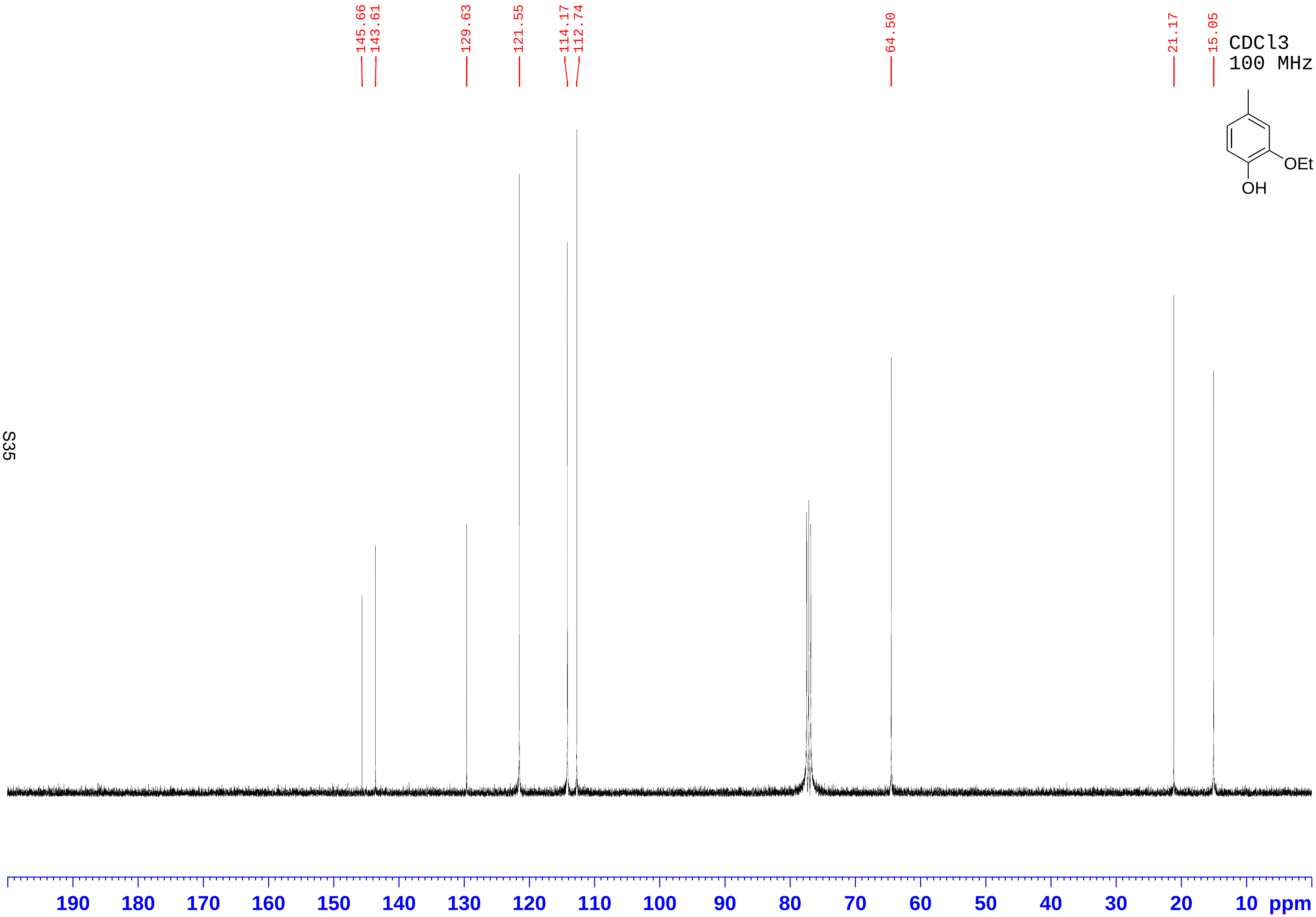

145.66
143.61
129.63
121.55
114.17
112.74
64.50
21.17
15.05
CDCl3
100 MHz
OEt
OH
190
180
170
160
150
140
130
120
110
100
90
80
70
60
50
40
30
20
10 ppm

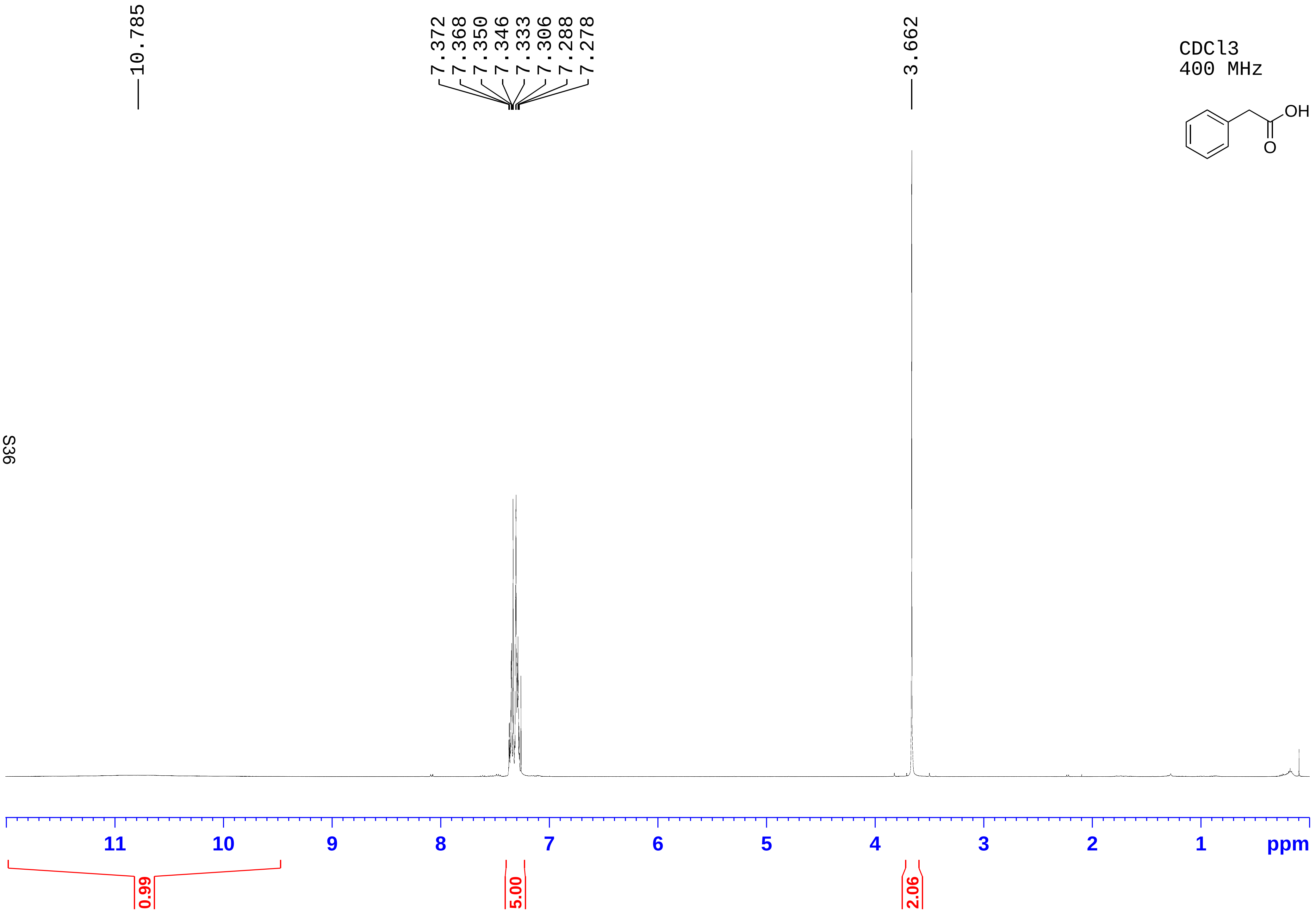

10.785
7.372
7.368
7.350
7.346
7.333
7.306
7.288
7.278
3.662
CDCl3
400 MHz
OH
O
11
10
9
8
7
6
5
4
3
2
1
ppm
0.99
5.00
2.06

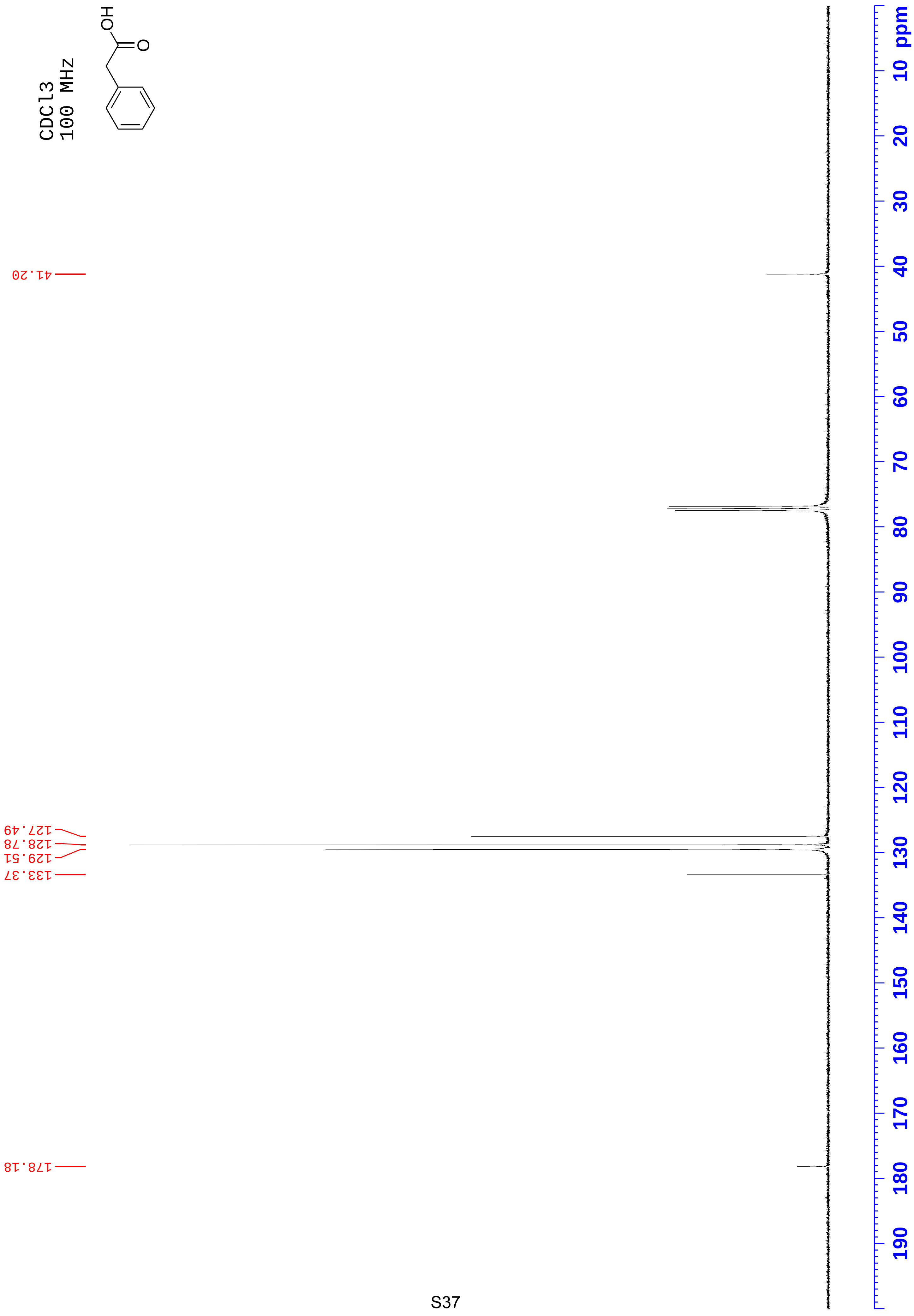
CDCl3
100 MHz
OH
O
41.20
127.49
128.78
129.51
133.37
178.18
ppm
10
20
30
40
50
60
70
80
90
100
110
120
130
140
150
160
170
180
190

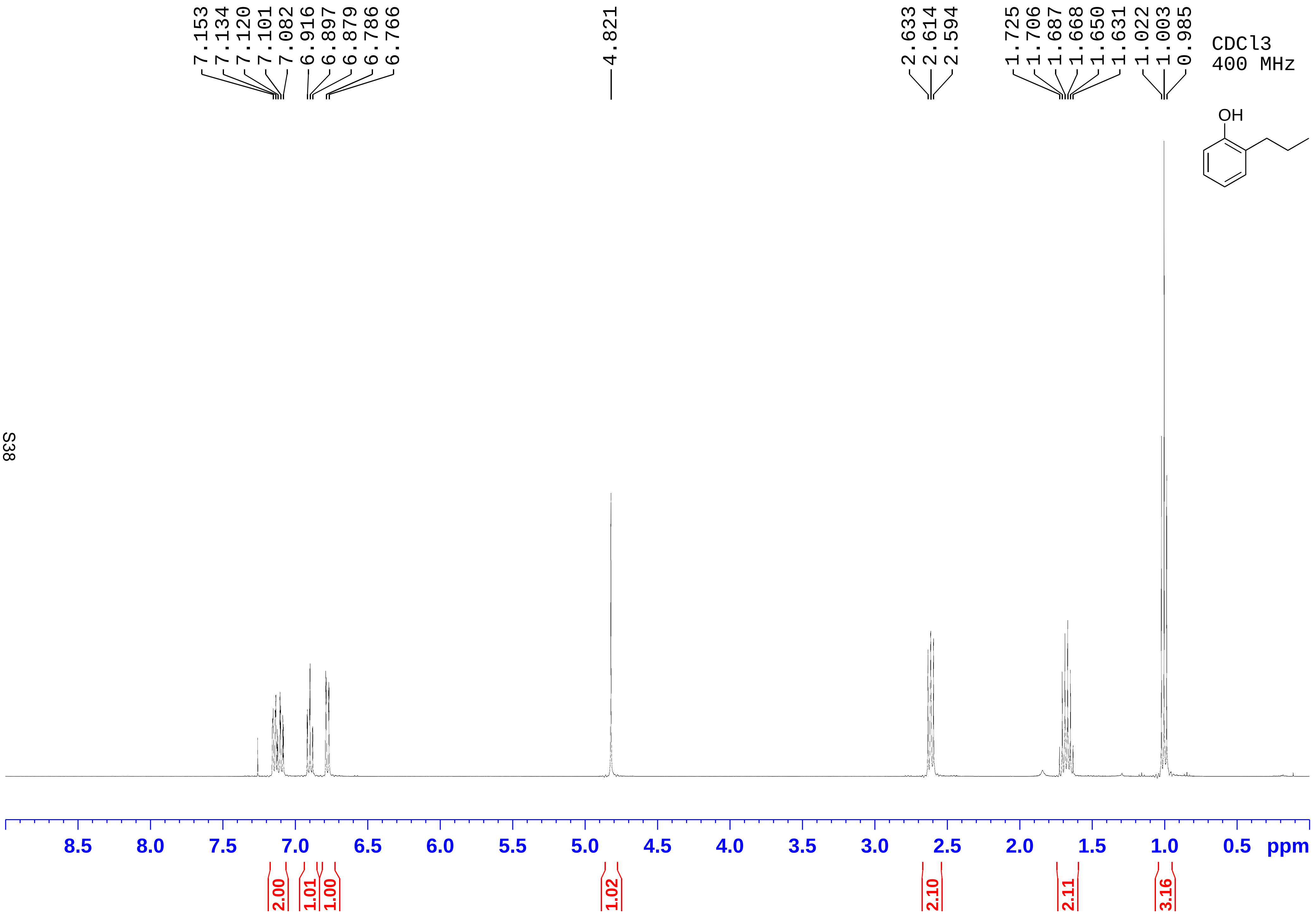

7.153
7.134
7.120
7.101
7.082
6.916
6.897
6.879
6.786
6.766
4.821
2.633
2.614
2.594
1.725
1.706
1.687
1.668
1.650
1.631
1.022
1.003
0.985
CDCl3
400 MHz
OH
8.5
8.0
7.5
7.0
6.5
6.0
5.5
5.0
4.5
4.0
3.5
3.0
2.5
2.0
1.5
1.0
0.5
ppm
2.00
1.01
1.00
1.02
2.10
2.11
3.16

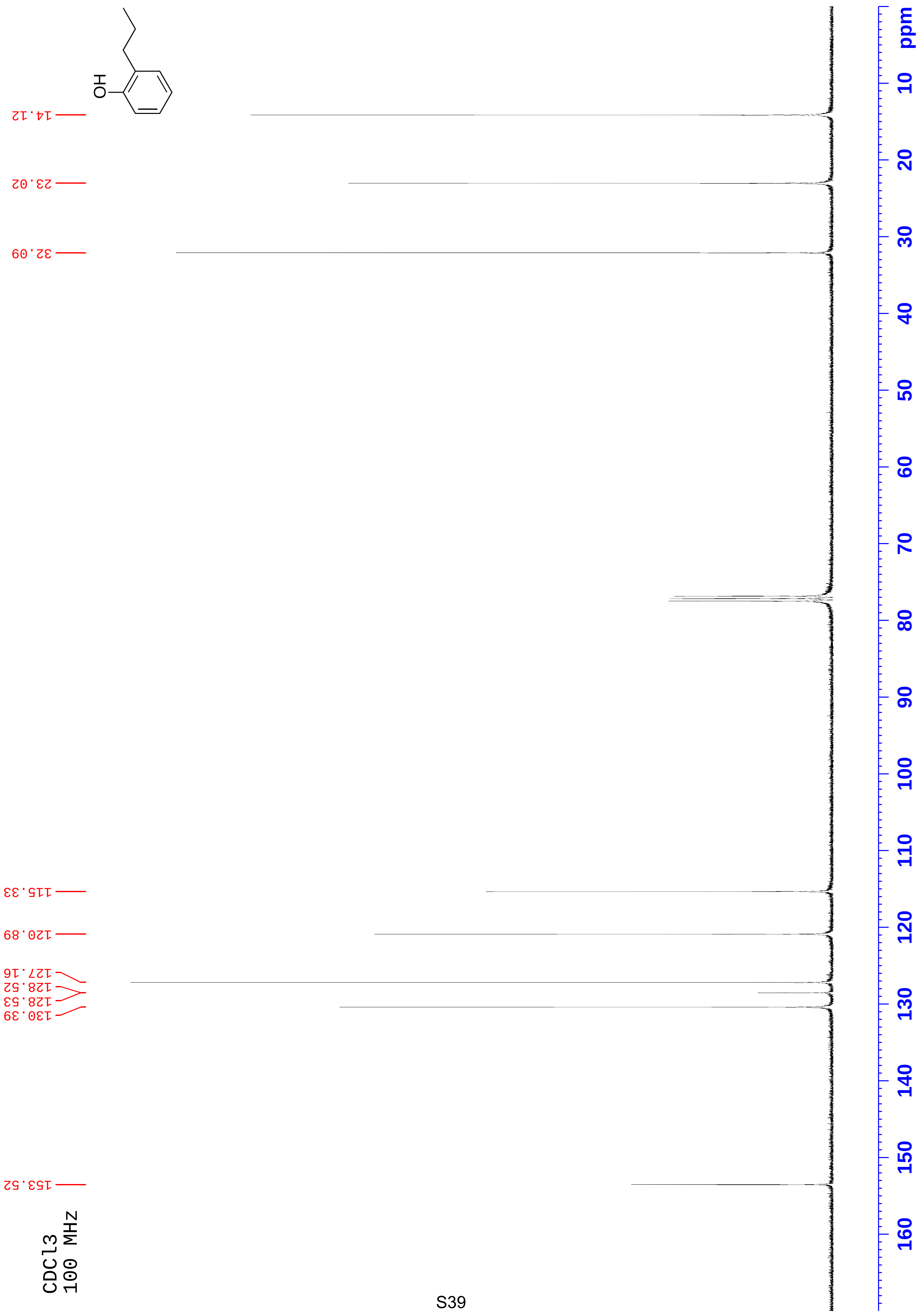
OH
14.12
23.02
32.09
115.33
120.89
127.16
128.52
128.53
130.39
153.52
CDCl3
100 MHz
ppm
10
20
30
40
50
60
70
80
90
100
110
120
130
140
150
160

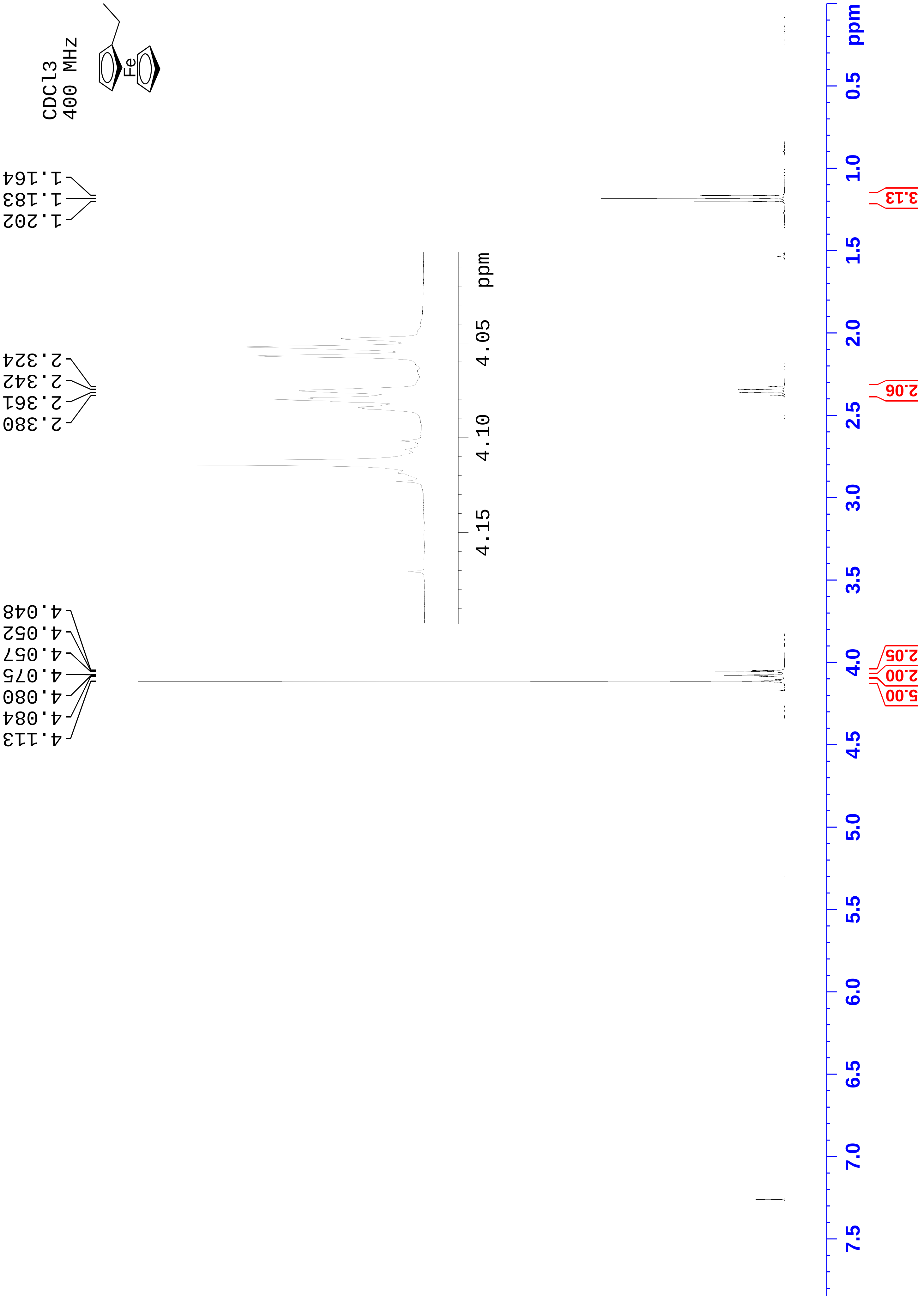
CDCl3
400 MHz
Fe
1.164
1.183
1.202
2.324
2.342
2.361
2.380
4.048
4.052
4.057
4.075
4.080
4.084
4.113
ppm
4.05
4.10
4.15
3.13
2.06
2.05
2.00
5.00
ppm
0.5
1.0
1.5
2.0
2.5
3.0
3.5
4.0
4.5
5.0
5.5
6.0
6.5
7.0
7.5

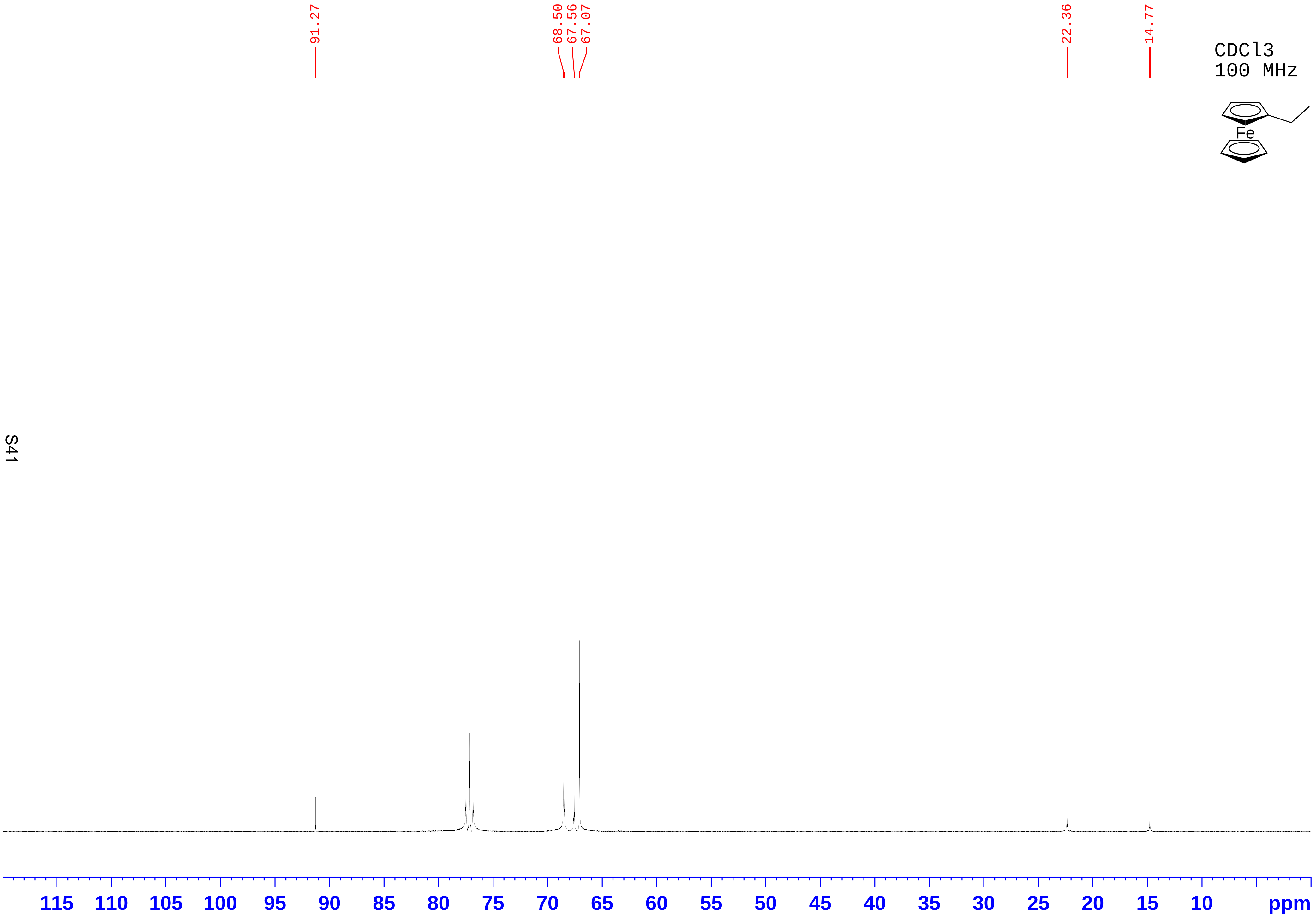
91.27
68.50
67.56
67.07
22.36
14.77
CDCl3
100 MHz
Fe
115 110 105 100 95 90 85 80 75 70 65 60 55 50 45 40 35 30 25 20 15 10 ppm

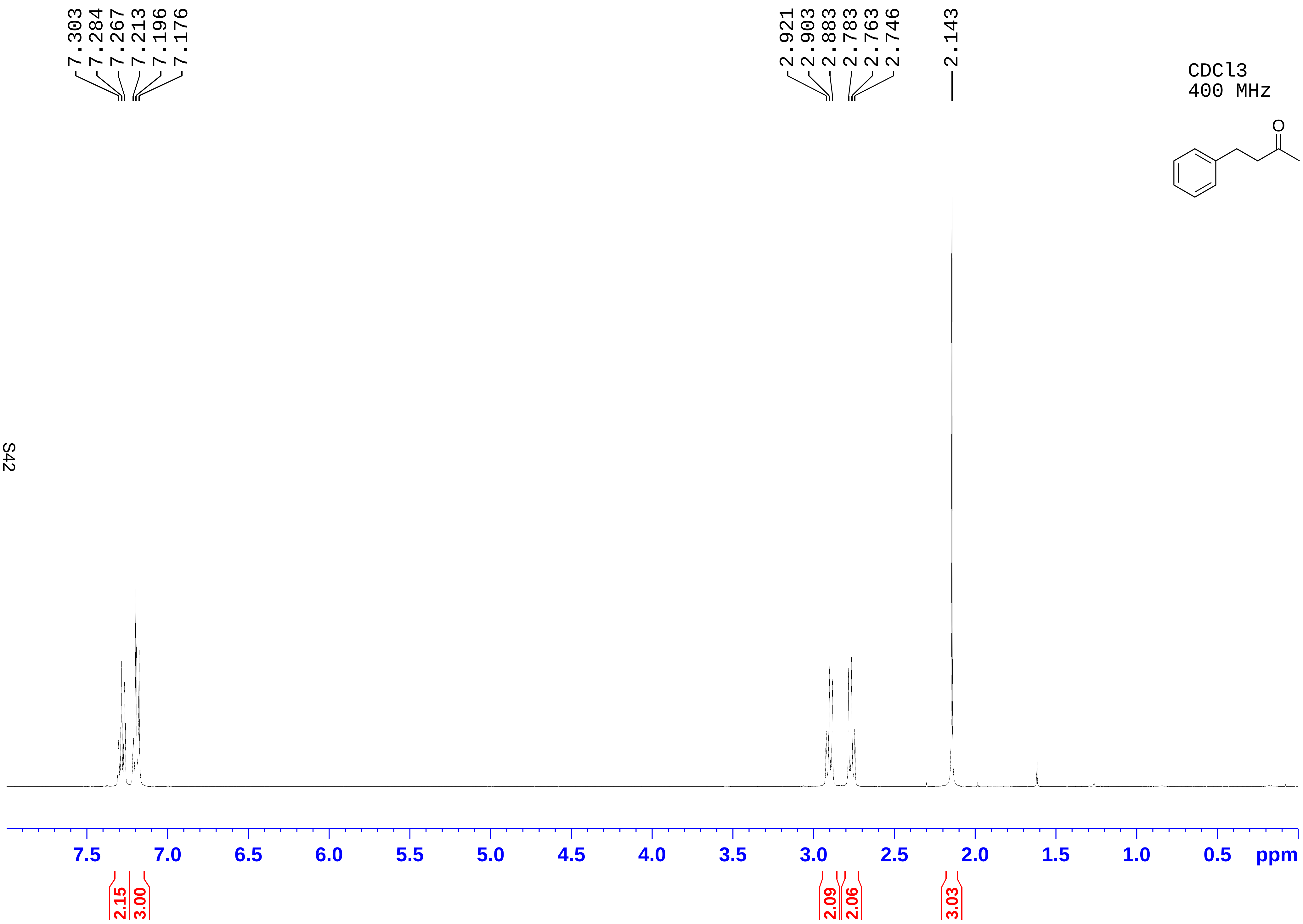

CDCl3
400 MHz
O
7.303
7.284
7.267
7.213
7.196
7.176
2.921
2.903
2.883
2.783
2.763
2.746
2.143
7.5
7.0
6.5
6.0
5.5
5.0
4.5
4.0
3.5
3.0
2.5
2.0
1.5
1.0
0.5
ppm
2.15
3.00
2.09
2.06
3.03

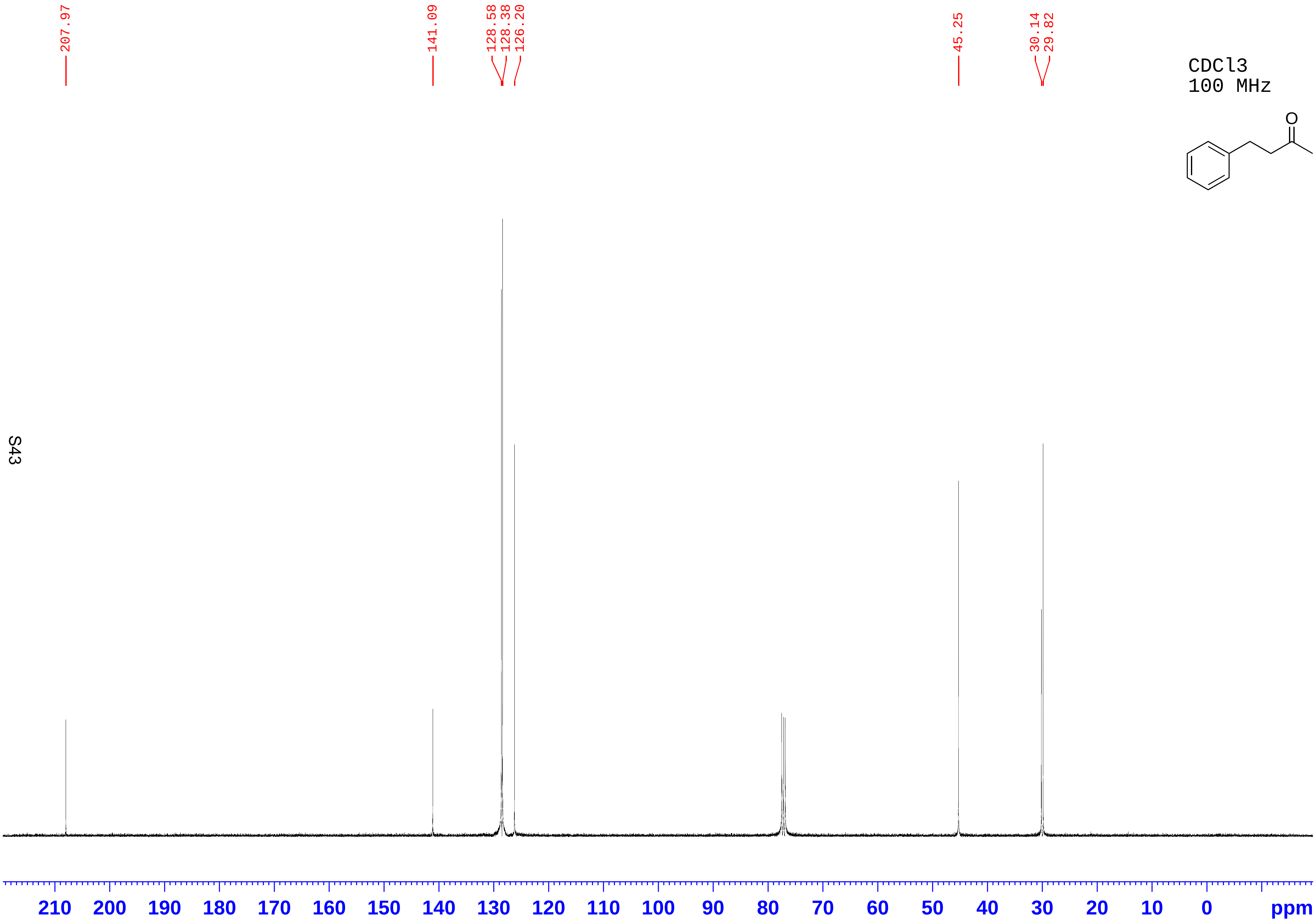
207.97
141.09
128.58
128.38
126.20
45.25
30.14
29.82
CDCl3
100 MHz
O
210 200 190 180 170 160 150 140 130 120 110 100 90 80 70 60 50 40 30 20 10 0 ppm
